# Plant-Derived Natural Products and Selective Apoptosis: A Cancer-Cell Vulnerability-State Framework from Redox Imbalance to Membrane–Ion Dysregulation

**DOI:** 10.3390/ph19081233

**Published:** 2026-08-05

**Authors:** Nurzhanyat Ablaikhanova, Gulmira Assan, Ranokhon Kurbannazarova, Botagoz Ussipbek, Arailym Yessenbekova, Akzhunis Zhumash, Aziza Bekenova, Marzhan Kulbayeva, Beibarys Mukhitdin, Aidos Bolatov

**Affiliations:** 1 Department of Biophysics, Biomedicine and Neuroscience, Farabi University, Almaty 050040, Kazakhstan; nurzhanat.ablaihanova@kaznu.kz (N.A.); gulmiraasan28@gmail.com (G.A.); arailym.yesenbekova@kaznu.kz (A.Y.); akzhunis.sagatbekovna@gmail.com (A.Z.); 2 Department of Normal Physiology with a Course of Biophysics, S.D. Asfendiyarov Kazakh National Medical University, Almaty 050012, Kazakhstan; bekenova-aziza@mail.ru; 3 Laboratory of Molecular Physiology, Institute of Biophysics and Biochemistry, National University of Uzbekistan, Tashkent 100174, Uzbekistan; rano_1971@mail.ru; 4 Laboratory of Environmental Physiology of Humans and Animals, Institute of Genetics and Physiology, Almaty 050060, Kazakhstan; mukhitdin00beibarys@gmail.com; 5 Guangdong Key Laboratory for Genome Stability & Disease Prevention and Carson International Cancer Center, Marshall Laboratory of Biomedical Engineering, Shenzhen University Medical School, Shenzhen University, Shenzhen 518037, China; bolatovaidos@gmail.com; 6 Department of Science, “University Medical Center” Corporate Fund, Astana 010000, Kazakhstan

**Keywords:** natural products, apoptosis, phytochemicals, anticancer therapeutics, anticancer efficacy, targeted cancer therapy, redox imbalance, mitochondrial priming, membrane–ion dysregulation

## Abstract

Plant-derived natural products remain a major source of anticancer chemical diversity, yet much of the preclinical literature still describes their activity through compound-centered readouts such as IC_50_ values, reactive oxygen species generation, mitochondrial depolarization, BCL-2-family remodeling, caspase activation, and PI3K/Akt or NF-κB inhibition. Although informative, these mechanisms do not fully explain why the same phytochemical induces apoptosis in some cancer cells, and cytostasis or adaptation in others, while normal cells frequently activate cytoprotective responses. Rather than replacing conventional compound-centered approaches, we integrate established concepts into a complementary vulnerability-state framework for interpreting plant-derived natural-product-induced selective apoptosis. In this framework, selective apoptosis occurs when phytochemical-induced stress intersects with pre-existing cancer-cell vulnerabilities, overwhelms adaptive buffering, and remains below the injury threshold of normal cells. We organize current evidence around redox imbalance, mitochondrial priming, membrane remodeling, ion-channel and cell-volume dysregulation, and survival-pathway addiction, emphasizing that pathway modulation becomes mechanistically meaningful when linked to differential cellular sensitivity. We further highlight membrane–ion biology as an underexplored contributor to phytochemical responses and discuss how chemical standardization, orthogonal cell-death assays, matched normal-cell models, organoids, and pharmacokinetic/pharmacodynamic considerations can advance the field from descriptive cytotoxicity toward mechanism-informed phytopharmacology.

## 1. Introduction

### 1.1. Why Natural Product Oncology Needs Reframing

Plant-derived natural products have long contributed to anticancer drug discovery and remain an important source of chemically diverse scaffolds for cancer prevention, therapy, and adjuvant treatment. For clarity, this review distinguishes several conceptually distinct classes of plant-derived materials, including chemically characterized phytochemicals, standardized botanical extracts, major bioactive constituents, semisynthetic or natural-product-derived derivatives, and clinically approved natural-product-derived anticancer drugs. These classes differ substantially in chemical identity, reproducibility, pharmacokinetics, regulatory maturity, and translational relevance. Accordingly, mechanistic interpretation throughout this review is made in the context of the specific material under investigation rather than treating all plant-derived agents as a homogeneous class. Evidence derived from crude botanical extracts, dietary exposures, and metabolites is interpreted more cautiously because of the additional challenges associated with chemical standardization, exposure assessment, and attribution of biological activity. Throughout this review, plant-derived natural products are used as the broad umbrella term encompassing chemically characterized phytochemicals, standardized botanical extracts, major bioactive constituents, semisynthetic or natural-product-derived derivatives, and approved natural-product-derived drugs. By contrast, phytochemicals refer specifically to naturally occurring plant-derived chemical constituents and should not be interpreted as synonymous with all plant-derived natural products.

Despite extensive chemical diversity, much of the literature remains compound-centered and descriptive. Studies typically report half-maximal inhibitory concentration (IC_50_), reactive oxygen species (ROS) generation, mitochondrial membrane depolarization, BAX/BCL-2 modulation, PI3K/Akt inhibition, or reduced proliferation after exposure to a phytochemical or extract. Although informative, these observations frequently describe downstream molecular events rather than explaining why certain cancer cells are selectively susceptible. Reviews of flavonoids, curcumin-related compounds, PI3K-modulating phytochemicals, and other plant-derived anticancer agents illustrate this emphasis on canonical apoptosis and signaling pathways, whereas the biological determinants of selectivity, sensitivity, and resistance remain less well defined [1,2,3,4,5].

Current evidence spans chemically heterogeneous materials, including purified phytochemicals, standardized botanical extracts, dietary constituents, metabolites, optimized derivatives, and approved natural-product-derived drugs. These categories differ substantially in chemical identity, reproducibility, pharmacokinetics, and translational maturity. Heavy reliance on viability assays and IC_50_ values often fails to distinguish cytostasis from apoptosis, necrosis, ferroptosis, or assay-related artifacts. Viability measurements are influenced by mitochondrial metabolism, membrane integrity, redox state, assay chemistry, cell density, and exposure time; therefore, a single metabolic assay cannot establish the mechanism of cell death [6]. In addition, apparent activity may result from assay interference, aggregation, impurities, nonspecific chemical reactivity, or unstable compounds rather than genuine biological effects [7,8]. Importantly, plant-derived compounds should not be regarded as inherently safer than synthetic anticancer agents. Their therapeutic value depends on the same principles that govern all anticancer therapeutics, including a favorable therapeutic window, reproducible chemical composition, pharmacokinetic suitability, and preferential activity against malignant rather than normal cells. Studies incorporating matched non-malignant cells and selectivity-index analyses provide stronger translational evidence by distinguishing preferential cancer-cell sensitivity from general cytotoxicity [9,10]. Nevertheless, many reports lack appropriate normal-cell controls, immune-cell compatibility studies, or organ-relevant toxicity models. These limitations are particularly relevant for membrane-active compounds such as saponins and for crude botanical extracts, which require rigorous botanical authentication, chemical fingerprinting, and batch-to-batch reproducibility before biological conclusions can be interpreted confidently [11,12,13,14,15].

These limitations also help explain why the same plant-derived compound or botanical preparation may produce different biological outcomes across experimental models. A compound may induce apoptosis in one cancer type, growth arrest in another, and adaptive or cytoprotective responses in normal cells. Such variability reflects differences in the biological state of the target cell rather than experimental noise. Cancer cells commonly exhibit elevated basal oxidative stress, mitochondrial priming, membrane remodeling, dysregulated ion homeostasis, and dependence on oncogenic survival pathways, creating vulnerabilities that are generally less pronounced in normal tissues [16,17,18,19,20,21]. Consequently, identical phytochemical exposure may remain below the stress threshold of normal cells while overwhelming adaptive capacity in susceptible cancer cells. Conventional compound-centered and target-based approaches remain indispensable for pharmacological characterization, but they do not fully explain these context-dependent responses.

Accordingly, we propose a complementary vulnerability-state framework for interpreting natural-product-induced selective apoptosis. Rather than focusing exclusively on molecular targets or signaling pathways altered by phytochemicals, this framework emphasizes pre-existing cancer-cell states that determine susceptibility to stress. We consider five principal vulnerability states: redox imbalance, mitochondrial priming, membrane remodeling, membrane–ion dysregulation (including altered ion-channel activity and cell-volume regulation), and survival-pathway addiction. Together, these states determine whether phytochemical exposure culminates in selective apoptosis or instead results in adaptive survival, cytostasis, ferroptosis, or non-selective toxicity, which are discussed only insofar as they inform the mechanisms and boundaries of selective apoptosis. Among them, membrane–ion dysregulation remains comparatively underexplored despite increasing evidence that ion flux, membrane potential, water transport, and apoptotic volume decrease (AVD) may contribute to apoptotic commitment.

The central conceptual contribution of this review is the vulnerability-state model of natural-product-induced selective apoptosis. In this model, selective apoptosis arises when phytochemical-induced stress intersects with pre-existing cancer-cell vulnerabilities, exceeds adaptive buffering capacity, and remains below the toxicity threshold of matched normal cells. This relationship is summarized by the following qualitative conceptual heuristic:

Selective apoptosis = phytochemical stress × pre-existing vulnerability × insufficient adaptive buffering × normal-cell sparing.(1)

Equation (1) is intended solely as a conceptual framework rather than a quantitative or predictive mathematical model. Phytochemical stress represents redox, mitochondrial, membrane, ion-homeostatic, metabolic, or survival-pathway perturbations induced by chemically characterized plant-derived compounds or standardized botanical preparations. Pre-existing vulnerability refers to biological features that place cancer cells closer to the threshold for irreversible death, including elevated basal ROS, mitochondrial priming, altered membrane composition, ion-channel dependence, impaired stress buffering, or addiction to PI3K/Akt, NF-κB, STAT3, or NRF2-associated survival signaling. Insufficient adaptive buffering reflects failure of antioxidant, autophagic, metabolic, proteostatic, or anti-apoptotic defense mechanisms. Normal-cell sparing defines selective apoptosis by requiring that equivalent exposure does not produce comparable lethal stress in matched non-malignant cells.

Using this framework, we organize the evidence according to cancer-cell vulnerability states rather than individual phytochemicals or isolated molecular targets. Although other regulated cell-death pathways are discussed where mechanistically relevant, they are considered primarily as alternative outcomes, adaptive responses, or differential mechanisms that influence the induction of selective apoptosis rather than as independent subjects of this review. We examine redox imbalance, mitochondrial priming, membrane remodeling, membrane–ion dysregulation (including altered ion-channel activity and cell-volume regulation), and survival-pathway addiction as determinants of selective apoptosis, while also discussing circumstances in which selectivity fails, including non-selective toxicity, hormesis, and regulated death programs other than apoptosis. By integrating mechanistic cell-death biology with phytochemical standardization and translational considerations, this review aims to shift natural-product oncology from descriptive cytotoxicity toward a mechanism-based understanding of selective anticancer activity.

Importantly, the proposed vulnerability-state framework does not introduce new mechanisms of cancer-cell death. Concepts such as mitochondrial priming, redox adaptation, oncogenic survival-pathway dependence, stress-buffer dependence, therapeutic windows, and cancer-cell vulnerability are already well established. The novelty of this Review does not lie in proposing new biological mechanisms of selective apoptosis. Rather, it lies in integrating established concepts—including oncogene addiction, non-oncogene addiction, synthetic lethality, stress-response dependence, mitochondrial priming, and redox adaptation—into a unified vulnerability-state framework for interpreting plant-derived natural-product-induced selective apoptosis.

### 1.2. Literature Search Strategy

This Review was conducted as a narrative, concept-driven literature review designed to synthesize current evidence supporting a vulnerability-state framework for plant-derived natural-product-induced selective apoptosis rather than to perform a formal systematic review or scoping review. Literature was primarily identified through searches of PubMed/MEDLINE, Web of Science, and Scopus, supplemented by citation tracking of relevant articles. Searches focused mainly on publications from approximately 2000 to June 2026, while seminal earlier studies were included where necessary to provide historical or mechanistic context. Representative search terms included combinations of plant-derived natural products, phytochemicals, botanical extracts, natural-product-derived drugs, apoptosis, selective apoptosis, cancer, ROS, redox imbalance, mitochondrial priming, membrane remodeling, ion channels, cell-volume regulation, survival signaling, ferroptosis, and related terms.

Priority was given to peer-reviewed studies providing mechanistic evidence, particularly investigations including matched normal-cell controls, orthogonal cell-death assays, chemically characterized compounds, pharmacokinetic or translational relevance, and studies using clinically relevant in vivo, organoid, or patient-derived models where available. Studies based solely on crude botanical extracts without adequate chemical characterization, insufficient methodological detail, or limited mechanistic evidence were interpreted cautiously and are discussed primarily in the context of the challenges associated with extract standardization. Rather than comprehensively cataloging all phytochemicals, the literature was synthesized thematically to develop and illustrate the proposed vulnerability-state framework.

Evidence grading in the major tables was qualitative and was used to distinguish the maturity of evidence rather than to provide a formal systematic review or assessment using the Grading of Recommendations Assessment, Development and Evaluation (GRADE) framework. “Clinically validated” indicates support from approved natural-product-derived anticancer drugs or relevant human clinical evidence. “Advanced preclinical” indicates support from in vivo, organoid, patient-derived, or other physiologically relevant models. “Preclinical” indicates evidence derived predominantly from conventional cancer-cell models, with or without limited in vivo confirmation. “Mechanistic” indicates that the proposed biological relationship has been supported by pathway-specific pharmacological or genetic manipulation, although translational validation remains limited. “Emerging/mechanistic inference” denotes preliminary or indirect evidence without sufficient causal validation, whereas “hypothesis-generating” or “hypothetical/underexplored” identifies proposed relationships for which direct natural-product-specific evidence remains sparse. Evidence levels refer to the highest level of support available for the stated relationship and should not be interpreted as applying uniformly to every listed compound or phytochemical class.

## 2. From Compound-Centered Pharmacology to Vulnerability-Centered Phytopharmacology

Natural-product oncology has traditionally been organized around a compound-centered question: what does compound X do to cancer cell line Y? This approach has generated extensive evidence that phytochemicals reduce viability, regulate apoptosis-related signaling, and modulate multiple stress-response pathways. However, these observations alone do not explain selective apoptosis. Many reviews already categorize plant-derived compounds according to canonical molecular pathways, whereas the more important translational question is why the same phytochemical induces apoptosis in some cancer cells but leads to resistance, cytostasis, alternative death programs, or nonspecific toxicity in others [1,2,3,4]. This limitation is particularly relevant because plant-derived natural products frequently function as multi-target biological stress modulators rather than single-target agents [5,22].

**Figure 1:**
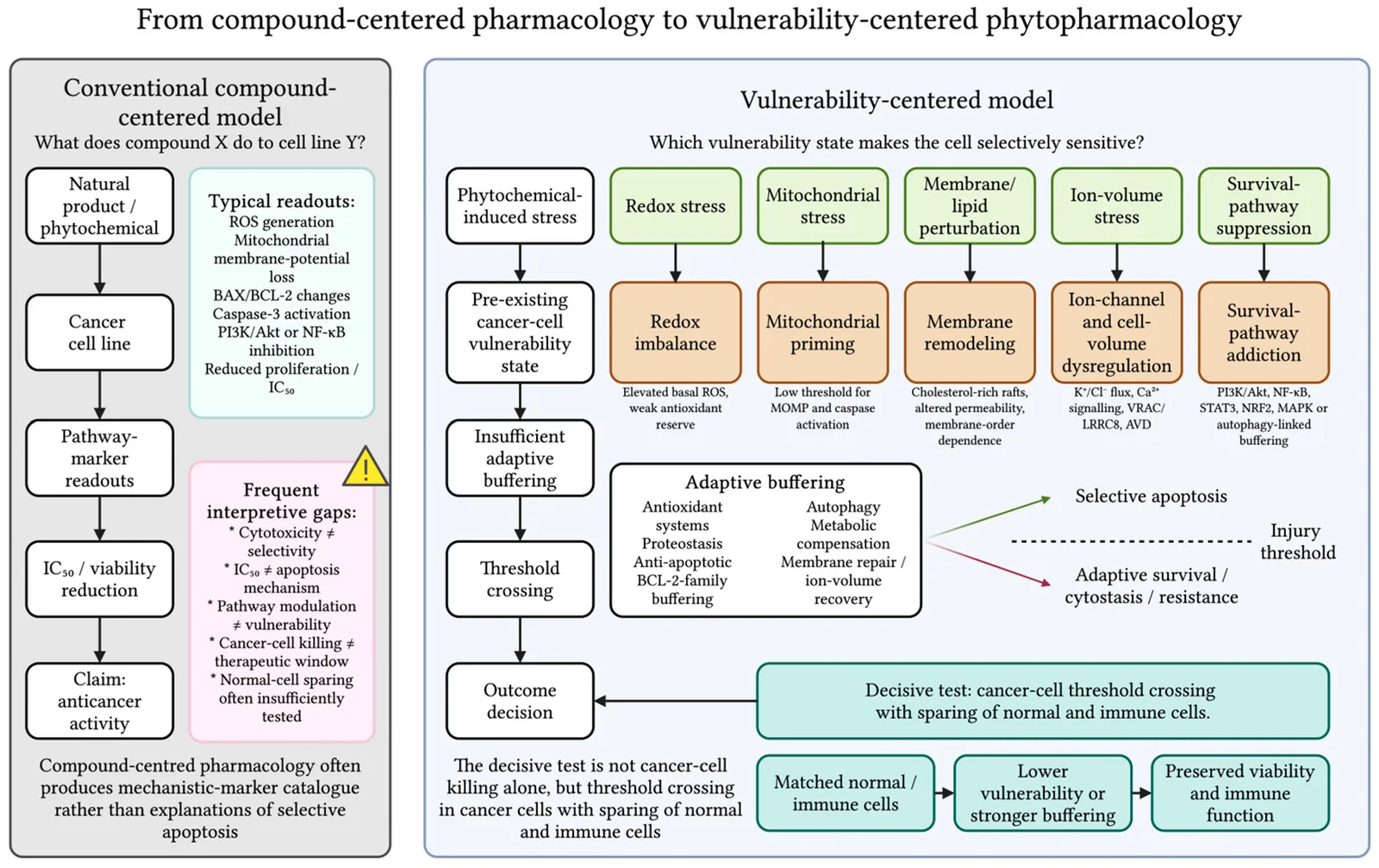
Conceptual transition from compound-centered pharmacology to vulnerability-centered phytopharmacology. Conventional compound-centered approaches primarily describe molecular responses following phytochemical exposure, whereas the proposed vulnerability-state framework emphasizes the interaction between phytochemical-induced stress and pre-existing cancer-cell vulnerabilities that enable selective apoptosis while sparing matched normal cells. Created in BioRender. Asanova, A. (2026) https://BioRender.com/5bh7fh5 (accessed on 2 August 2026).

Accordingly, we propose a vulnerability-centered framework as the organizing principle of this Review (Figure 1). Rather than asking only what changes a phytochemical induces after exposure, this framework asks which pre-existing cancer-cell vulnerabilities determine susceptibility to phytochemical-induced stress and whether that stress exceeds the cancer-cell death threshold while remaining below the toxicity threshold of normal cells. Within this framework, the term cancer-cell vulnerability architecture refers to the integrated configuration of pre-existing biological vulnerabilities that collectively determines a cell’s susceptibility to phytochemical-induced stress. Rather than representing a single molecular alteration, this architecture encompasses the combined state of redox imbalance, mitochondrial priming, membrane remodeling, membrane–ion dysregulation, survival-pathway addiction, and the capacity of adaptive buffering systems. Differences in this vulnerability architecture explain why identical phytochemical exposure may produce selective apoptosis in one cellular context but adaptive survival, cytostasis, or alternative outcomes in another. A vulnerability state is defined as a pre-existing biological condition that lowers the threshold for cell death following exposure to a stressor. In natural-product oncology, these states include redox imbalance, mitochondrial priming, membrane remodeling, ion-channel and cell-volume dependence, survival-pathway addiction, and impaired adaptive buffering. This perspective shifts phytopharmacology from describing molecular responses toward explaining the biological conditions that determine selective susceptibility.

Within this framework, selective apoptosis is defined as the preferential induction of apoptosis in malignant cells relative to biologically relevant matched non-malignant cells under comparable exposure conditions. It emerges through the interaction of four interconnected components: phytochemical-induced stress, pre-existing cancer-cell vulnerability, insufficient adaptive buffering, and normal-cell sparing. Differential sensitivity among cancer cell lines or molecular subtypes is interpreted as evidence of differences in cancer-cell vulnerability architecture rather than as evidence of selective apoptosis by itself. Such comparisons are valuable for identifying vulnerability states but should ideally be complemented by appropriate normal-cell controls when therapeutic selectivity is inferred.

Rather than treating individual molecular changes as evidence of efficacy by themselves, the proposed framework interprets these changes within the biological context of the target cell. It does not replace established concepts such as mitochondrial priming, oncogene addiction, synthetic lethality, or redox adaptation; instead, it integrates them into a unified conceptual model for interpreting plant-derived natural-product-induced selective apoptosis. Consequently, structurally diverse phytochemicals can be understood as converging on common vulnerability states despite acting through different molecular targets [16,23].

A central distinction is the difference between a mechanism and a vulnerability state. A mechanism describes the biological changes that occur after phytochemical exposure, whereas a vulnerability state explains why a cell was predisposed to respond before exposure. Molecular responses therefore become evidence of vulnerability only when they explain differential sensitivity between malignant and non-malignant cells or between sensitive and resistant cancer models. This distinction shifts interpretation from descriptive pathway reporting to biologically meaningful explanations of selective susceptibility.

The framework also reframes selectivity. Plant-derived compounds are not selectively cytotoxic because they function as intrinsically “smart” therapeutics, but because some cancer cells exist closer than normal cells to a critical threshold of biological failure. Selective apoptosis therefore reflects the interaction between phytochemical-induced stress and cancer-specific vulnerability rather than compound activity alone. Cancer cells should be viewed not as passive targets but as biologically primed systems with distinct vulnerability architectures that determine their stress tolerance [11,20,21,24].

A vulnerability-centered approach also strengthens translational relevance by emphasizing the therapeutic window rather than cancer-cell cytotoxicity alone. Selective activity should be demonstrated through appropriate normal-cell comparisons, selectivity-index calculations, chemically defined test materials, and reproducible mechanistic validation [9,10,12]. This emphasis is essential because apparent activity may be influenced by assay artifacts, extract variability, impurities, nonspecific chemical reactivity, or unstable compounds [7,8,13]. Accordingly, apoptosis is interpreted not as an isolated endpoint after compound exposure but as the outcome of a state-dependent interaction among stress induction, adaptive buffering capacity, and normal-cell tolerance.

Importantly, the proposed vulnerability states should not be regarded as universal or static properties shared equally across all cancers. Their relative importance is expected to differ among tumor types, molecular subtypes, and stages of disease progression according to the underlying genetic, epigenetic, metabolic, and microenvironmental context. Moreover, therapeutic pressure may reshape these vulnerability states during disease evolution, resulting in altered biological dependencies in treatment-resistant populations. For example, resistance to chemotherapy or targeted therapies is frequently accompanied by metabolic reprogramming, enhanced antioxidant buffering, survival-pathway rewiring, and alterations in membrane organization or ion-homeostatic regulation, potentially creating new therapeutic vulnerabilities. Accordingly, the proposed framework should be interpreted as a context-dependent model in which the dominant vulnerability state varies across biological and clinical settings rather than as a fixed hierarchy applicable to all cancers. This perspective also provides a conceptual basis for biomarker-guided patient stratification and the identification of context-specific opportunities for vulnerability-directed phytopharmacological intervention.

Throughout this review, we use this vulnerability-centered framework to integrate the major biological determinants of selective apoptosis into a unified interpretation of natural-product action. Rather than asking only which phytochemicals affect cancer cells, we ask which cancer-cell vulnerability states permit selective apoptotic death while preserving normal-cell function. This reframing shifts natural-product oncology from compound-centered cytotoxicity toward mechanism-based phytopharmacology.

The proposed vulnerability-state framework is complementary to, rather than a replacement for, established concepts in cancer biology. Oncogene addiction describes dependence on specific oncogenic signaling pathways; non-oncogene addiction emphasizes reliance on non-mutated stress-support pathways; synthetic lethality refers to cell death resulting from defined combinations of genetic perturbations; and stress-response vulnerabilities describe dependence on adaptive systems that maintain cellular homeostasis. Rather than introducing new biological mechanisms or a new therapeutic paradigm, the proposed vulnerability-state framework may contribute an integrative, phytopharmacology-oriented model for interpreting plant-derived natural-product-induced selective apoptosis. It organizes these complementary concepts according to the pre-existing biological state of the cancer cell and explains how diverse phytochemical-induced stresses converge on common vulnerability states to determine selective apoptotic responses. To further clarify this conceptual contribution, Table 1 summarizes the relationship between the proposed framework and these established concepts.

## 3. Plant-Derived Compounds as Multi-Target Stress Modulators

### 3.1. Multi-Target Biology

Plant-derived natural products should not be viewed primarily as single-target cytotoxins. Although target deconvolution remains essential for drug development, many phytochemicals act as multi-target modulators of interconnected stress-response networks rather than isolated molecular pathways. Their biological effects therefore depend less on compound identity than on the vulnerability state of the target cell. Throughout this review, representative phytochemicals and other chemically characterized plant-derived compounds are used not as comprehensive examples of anticancer activity, but as mechanistic illustrations of how distinct classes of plant-derived agents engage different vulnerability states.

This view is consistent with contemporary natural-product drug-discovery frameworks, which recognize that natural scaffolds commonly regulate interconnected signaling, metabolic, and cell-death networks [25]. Rather than functioning as weaker analogs of conventional drugs, phytochemicals modulate redox homeostasis, mitochondrial function, proteostasis, inflammatory signaling, membrane organization, and programmed cell-death pathways [26,27,28]. Their therapeutic relevance therefore depends on whether these effects expose biologically meaningful cancer-cell vulnerabilities while sparing non-malignant cells.

Because these adaptive systems are highly interconnected, identical phytochemicals may produce apoptosis, cytostasis, alternative regulated cell death, or adaptive survival depending on cellular context. Mitochondrial priming, basal oxidative stress, metabolic state, and crosstalk among apoptosis, autophagy, ferroptosis, pyroptosis, necroptosis, and paraptosis all influence cell fate following phytochemical exposure [26,27,28,29,30,31]. Consequently, mechanistic interpretation should focus on the biological state of the target cell rather than on isolated pathway modulation.

### 3.2. Membrane–Ion Biology as an Emerging Vulnerability Layer

In addition to redox and mitochondrial biology, this review highlights membrane–ion homeostasis as an emerging vulnerability layer. Ion flux, membrane potential, osmotic balance, and AVD are recognized components of apoptosis, yet their specific contribution to phytochemical-induced selective apoptosis remains incompletely understood. Because cancer cells frequently remodel ion channels, transporters, lipid rafts, membrane potential, and cell-volume regulation to support proliferation and therapy resistance, these adaptations may create vulnerabilities that complement redox imbalance and mitochondrial priming.

Plant-derived compounds may influence membrane-associated signaling, calcium homeostasis, potassium and chloride flux, aquaporin-dependent water transport, and the activity of volume-regulated anion channels (VRACs) formed by leucine-rich repeat-containing protein 8 (LRRC8) subunits [32]. Saponins currently provide the strongest evidence for membrane-directed activity through cholesterol-dependent membrane interactions, although these effects may also increase nonspecific toxicity. By contrast, direct evidence linking phytochemicals to VRAC/LRRC8-mediated AVD, aquaporin function, or other ion-volume regulatory mechanisms remains limited. Membrane–ion biology should therefore be regarded as an emerging mechanistic hypothesis requiring further experimental validation.

Future studies should establish whether membrane–ion alterations initiate apoptotic commitment or arise secondarily during cell death. Time-resolved analyses integrating ion flux, membrane potential, mitochondrial function, and apoptosis markers, together with pharmacological or genetic perturbation of candidate ion-transport pathways, will be essential for establishing causality. For membrane-active phytochemicals, selective apoptotic signaling must also be distinguished from nonspecific membrane injury by appropriate normal-cell controls and membrane-integrity assays.

### 3.3. Purified Compounds, Crude Extracts, and Translational Considerations

Mechanistic interpretation also depends on the nature of the test material. Many studies evaluate crude extracts or supraphysiological concentrations of purified compounds, whereas human exposure is determined by solubility, absorption, metabolism, microbiome transformation, plasma binding, and tissue distribution [25,33]. Consequently, neither plant-derived origin nor in vitro cytotoxicity should be interpreted as evidence of therapeutic potential or safety. Therapeutic relevance must instead be demonstrated through pharmacokinetic, toxicological, and cancer-selectivity studies.

Robust translational studies should identify the active chemical species, demonstrate target engagement at biologically relevant concentrations, compare malignant with matched non-malignant cells, and incorporate pharmacokinetic or metabolite data whenever possible. Without these elements, apparent selectivity may instead reflect extract variability, assay interference, chemical instability, or nonspecific toxicity rather than true therapeutic vulnerability.

Evidence derived from approved natural-product-derived anticancer drugs should not be interpreted interchangeably with evidence obtained from investigational phytochemicals, dietary constituents, or crude botanical extracts. The clinical success of approved natural-product-derived drugs cannot be extrapolated automatically to unrelated investigational compounds simply because they share a natural origin. Agents such as paclitaxel and the vinca alkaloids have undergone extensive medicinal chemistry optimization, pharmaceutical development, pharmacokinetic characterization, and rigorous clinical validation. By contrast, most phytochemicals remain at the preclinical stage, dietary constituents generally achieve substantially lower systemic exposures, and crude botanical extracts often contain multiple chemically undefined constituents with variable composition. Consequently, approved natural-product-derived drugs should be regarded as proof of the therapeutic potential of natural-product scaffolds rather than validation of unrelated investigational phytochemicals or botanical extracts. Mechanistic and translational confidence should therefore be interpreted in the context of the specific material under investigation rather than under the broad umbrella term plant-derived natural products.

### 3.4. Organizing Phytochemicals by Vulnerability States

Accordingly, Table 2 is not intended as a catalog of phytochemicals but as a framework illustrating how diverse natural-product classes can converge on common cancer-cell vulnerability states. Unlike conventional classifications based on chemical structure or molecular targets, this organization emphasizes the biological context that determines selective apoptosis.

**Figure 2:**
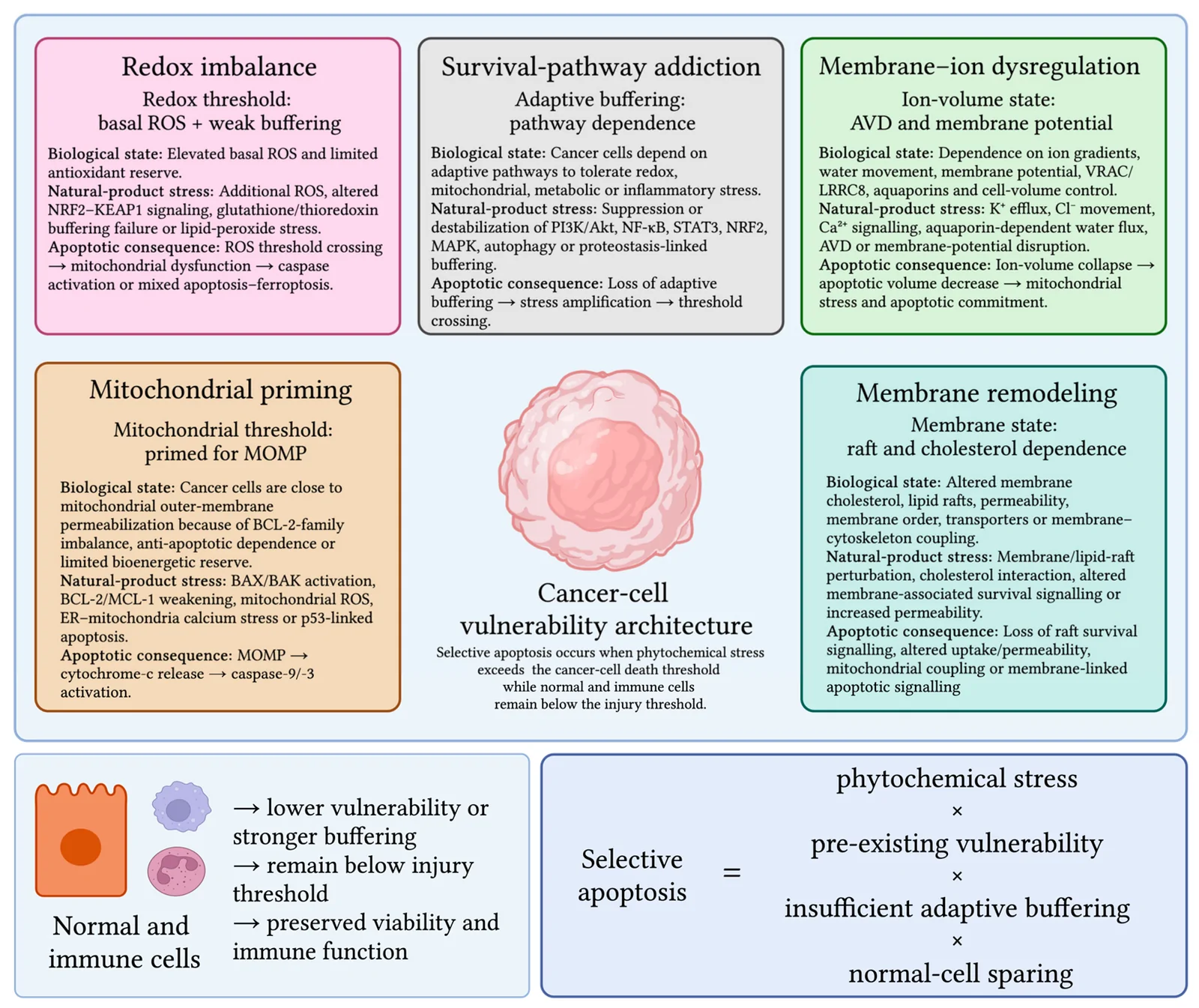
The five vulnerability states underlying natural-product-induced selective apoptosis. The proposed framework highlights five interconnected vulnerability states—redox imbalance, mitochondrial priming, membrane remodeling, membrane–ion dysregulation (including altered ion-channel activity and cell-volume regulation), and survival-pathway addiction—that collectively determine whether phytochemical-induced stress selectively triggers apoptosis in cancer cells. Created in BioRender. Asanova, A. (2026) https://BioRender.com/pznp5uv (accessed on 2 August 2026).

The biological activity of plant-derived compounds is inherently context-dependent. The same compound may produce cytostatic, cytotoxic, antioxidant, pro-oxidant, chemosensitizing, or protective effects depending on dose, exposure duration, cancer lineage, mutational background, mitochondrial function, baseline oxidative stress, extract composition, bioavailability, and metabolism. As summarized in Figure 2, selective apoptosis is best understood as the consequence of phytochemical-induced stress interacting with five interconnected vulnerability states—redox imbalance, mitochondrial priming, membrane remodeling, membrane–ion dysregulation (including altered ion-channel activity and cell-volume regulation), and survival-pathway addiction—while remaining below the injury threshold of normal cells. This vulnerability-state framework provides the conceptual basis for the biological sections that follow.

## 4. Vulnerability State I: Redox Imbalance

Redox imbalance is one of the best-established vulnerability states through which plant-derived natural products may promote selective apoptosis. Rather than being passive by-products of transformation, ROS support proliferation, metabolic adaptation, invasion, and survival signaling in many cancers. Because malignant cells frequently operate with chronically elevated basal ROS, they remain close to a critical redox threshold at which additional oxidative stress shifts signaling toward oxidative damage, mitochondrial dysfunction, and programmed cell death. Table 3 summarizes this vulnerability state as a network of interconnected redox-control modules rather than a single molecular mechanism [2,3,5,8,17,18,19,22,36,42,59,62,63,64,65,66,67,68,69,70,71,72].

Cancer-cell survival therefore depends on antioxidant-buffering systems, including glutathione, thioredoxin, peroxiredoxins, and NRF2-regulated detoxification pathways. NRF2 illustrates the context dependence of redox biology: transient activation protects normal tissues from oxidative injury, whereas persistent activation in established tumors may promote survival, metastasis, and therapeutic resistance [73,74]. Consequently, phytochemicals such as sulforaphane, curcumin, resveratrol, quercetin, EGCG, and berberine may either enhance antioxidant adaptation or overwhelm it depending on cellular context. Interpretation should therefore incorporate measurements of NRF2 activity, glutathione status, and antioxidant capacity rather than relying solely on ROS detection.

Mitochondria provide the principal link between redox imbalance and apoptosis. Excessive ROS can impair electron transport, oxidize mitochondrial lipids and proteins, collapse mitochondrial membrane potential, and trigger mitochondrial outer-membrane permeabilization (MOMP), leading to cytochrome c release and caspase-9/-3 activation [23,75]. Numerous phytochemicals, including berberine, curcumin, resveratrol, EGCG, quercetin, apigenin, and luteolin, have been reported to influence mitochondrial ROS, BCL-2-family signaling, membrane potential, and caspase activation. However, mitochondrial depolarization alone should not be considered definitive evidence of apoptosis without temporal association with oxidative stress and confirmation by antioxidant or mitochondrial-targeted rescue experiments.

Redox imbalance also intersects with DNA damage and lipid peroxidation. Oxidative DNA lesions activate DNA-damage responses and apoptosis, whereas lipid peroxidation may contribute to apoptosis, ferroptosis, or mixed regulated cell-death phenotype. Antioxidant regulators such as SLC7A11, GCLC, GPX4, thioredoxin, and thioredoxin reductase influence whether oxidative stress remains tolerable or becomes lethal [70]. Accordingly, lipid peroxidation should be interpreted together with caspase activation, ferroptosis-rescue experiments, and morphological criteria to define the predominant death program.

Additional complexity arises from NADPH oxidases and hypoxia. NOX-derived ROS and hypoxia-inducible factor (HIF) promote proliferation, angiogenesis, metabolic adaptation, and antioxidant responses. Plant-derived compounds may either suppress these tumor-promoting pathways or intensify oxidative stress until adaptive buffering fails, depending on the biological context [76,77].

Future studies should move beyond descriptive ROS measurements and define redox vulnerability using integrated functional analyses. Time-resolved assessment of ROS generation, antioxidant-buffering capacity, NRF2 activity, glutathione metabolism, mitochondrial dysfunction, and rescue by antioxidants or genetic perturbation will be essential for distinguishing adaptive redox signaling from lethal oxidative stress and for identifying the tumor contexts in which phytochemical-induced redox modulation is most likely to produce selective apoptosis.

## 5. Vulnerability State II: Mitochondrial Priming and Apoptotic Threshold

Mitochondrial priming helps explain why some cancer cells undergo apoptosis after relatively modest phytochemical stress, whereas non-malignant cells or less-primed tumors remain viable. Priming reflects the balance between pro-apoptotic pressure and anti-apoptotic buffering at the mitochondrial outer membrane. In highly primed cells, BAX and BAK are restrained by anti-apoptotic proteins, including BCL-2, BCL-xL, and MCL-1, yet the system remains close to MOMP. Consequently, relatively small perturbations can trigger cytochrome c release, apoptosome formation, and caspase-dependent apoptosis. Table 4 summarizes the major mitochondrial control points through which representative phytochemicals and other plant-derived compounds may lower this apoptotic threshold [2,3,5,22,36,42,62,78,79,80].

Mitochondrial apoptosis behaves as a threshold-governed process rather than a gradual decline in viability. Studies using BH3 profiling demonstrate that apoptotic sensitivity depends less on the absolute abundance of BCL-2-family proteins than on mitochondrial dependence on BCL-2, BCL-xL, or MCL-1 and the availability of sequestered BH3-only proteins [81,82]. This explains why phytochemicals with moderate biochemical effects may induce robust apoptosis in selected cancer contexts. When mitochondria are already highly primed, relatively modest shifts in BH3 signaling or anti-apoptotic buffering can be sufficient to activate BAX/BAK and initiate MOMP.

Many plant-derived compounds influence multiple components of mitochondrial stress simultaneously. Berberine, curcumin, quercetin, resveratrol, celastrol, triptolide, and ginsenosides have been reported to regulate mitochondrial membrane potential, ROS generation, BCL-2-family signaling, endoplasmic reticulum (ER) stress, p53-associated pathways, and caspase activation in experimental cancer models. Their significance lies not in acting as direct BH3 mimetics but in lowering the apoptotic threshold through coordinated disruption of mitochondrial homeostasis. In highly primed cells, these combined perturbations may be sufficient to cross the MOMP threshold, whereas less-primed cells may undergo only adaptive or cytostatic responses.

Mitochondrial bioenergetics further influences apoptotic susceptibility. Cancer cells with high respiratory demand, limited spare respiratory capacity, or impaired mitochondrial quality control may be particularly sensitive to agents that disrupt electron transport, membrane potential, or redox homeostasis [83,84]. Likewise, p53 status can modify mitochondrial priming because wild-type p53 promotes expression of BAX, PUMA, and NOXA, whereas mutant p53 may attenuate apoptosis or acquire pro-survival functions [85,86]. Nevertheless, mitochondrial apoptosis also occurs through p53-independent mechanisms, including ER stress, oxidative injury, calcium transfer, and direct remodeling of BCL-2-family proteins.

Demonstrating mitochondrial priming requires distinguishing a low apoptotic threshold from nonspecific mitochondrial toxicity. Mitochondrial depolarization, ATP depletion, and ROS accumulation are not sufficient by themselves because they also occur during necrosis, ferroptosis, and late-stage cell death. Stronger evidence is provided when time-resolved experiments demonstrate that BCL-2-family remodeling, BAX/BAK activation, and cytochrome c release precede caspase activation and membrane disruption. Complementary approaches, including BH3 profiling, BCL-2-family rescue experiments, BAX/BAK knockout, caspase inhibition, and comparison with matched non-malignant cells, are essential to establish that phytochemicals selectively exploit mitochondrial priming rather than causing nonspecific mitochondrial injury.

## 6. Vulnerability State III: Membrane Remodeling and Permeability

The plasma membrane is not merely a passive barrier to phytochemical uptake but a dynamic regulator of signaling, mechanotransduction, and stress adaptation. During malignant transformation, cancer cells remodel membrane lipid composition, cholesterol distribution, phospholipid asymmetry, membrane fluidity, lipid-raft architecture, transporter expression, and organelle-membrane interactions. These changes influence not only compound uptake but also the organization of survival and death signaling, making membrane remodeling a potential determinant of phytochemical sensitivity. Table 5 summarizes the principal membrane-related vulnerability modules relevant to natural-product-induced apoptosis [2,3,5,11,24,36,62,87,88,89,90,91,92,93].

Cancer cells frequently reprogram lipid metabolism, altering membrane order, sphingolipid composition, cholesterol content, and bilayer fluidity [94,95]. These changes affect receptor clustering, endocytosis, mechanotransduction, drug transport, and signaling efficiency. Because membrane composition varies among tumor types and disease stages, identical phytochemicals may partition differently into malignant and non-malignant membranes, contributing to context-dependent biological responses.

Lipid rafts provide one of the clearest examples of membrane-associated vulnerability. These cholesterol- and sphingolipid-rich microdomains organize signaling complexes, including PI3K–Akt, MAPK, NF-κB, Src-family kinases, integrins, and death receptors [96,97]. Their importance in proliferation, invasion, and therapeutic resistance may also create susceptibility to compounds that disrupt cholesterol organization or raft integrity. This concept is particularly relevant for amphipathic phytochemicals and saponins, although pathway inhibition alone does not demonstrate raft targeting. Robust evidence requires showing that raft disruption precedes downstream signaling changes, ideally using cholesterol-depletion and cholesterol-repletion experiments.

Membrane permeability is another important but more challenging vulnerability to interpret. Altered permeability may influence calcium influx, osmotic balance, and intracellular stress signaling, but it may also reflect nonspecific membrane injury. Accordingly, membrane-active phytochemicals should be evaluated using time-resolved assays that distinguish regulated cellular responses from primary membrane disruption. Likewise, phosphatidylserine externalization should be interpreted as an apoptosis-associated membrane event rather than direct evidence of selective targeting of cancer-cell membranes [98,99].

A further distinction should be made between plasma, mitochondrial, and lysosomal membrane responses. Mitochondrial membrane permeabilization promotes intrinsic apoptosis through cytochrome c release, whereas lysosomal membrane permeabilization releases cathepsins that can amplify mitochondrial apoptosis or initiate lysosome-dependent cell death [100,101]. Consequently, membrane-associated effects should be interpreted according to their subcellular localization and temporal sequence, as similar phenotypes may arise from distinct biological processes.

Membrane biology also influences pharmacological exposure. Many phytochemicals exhibit poor aqueous solubility, limited permeability, or active efflux through ATP-binding cassette (ABC) transporters, whereas lipid-based formulations may increase intracellular accumulation. Likewise, transporter modulation by curcumin, resveratrol, quercetin, berberine, piperine, or EGCG may enhance intracellular drug retention while also affecting systemic exposure. Uptake-normalized comparisons are therefore needed to distinguish improved delivery from genuine biological selectivity.

Finally, membrane safety requires careful evaluation. Hemolysis is a useful indicator of membrane toxicity for amphipathic compounds but cannot predict effects in nucleated cells because erythrocytes lack mitochondria and many stress-response pathways. A more informative safety assessment should combine hemolysis with matched non-malignant cell models and organ-relevant toxicity systems. Accordingly, membrane-associated effects should be classified as selective membrane-linked vulnerability, delivery-dependent exposure, or unacceptable membrane toxicity rather than being regarded as nonspecific by default.

## 7. Vulnerability State IV: Ion-Channel Dependence and Cell-Volume Dysregulation

Ion-channel dependence and cell-volume regulation represent one of the least developed and most hypothesis-generating vulnerability states in natural-product oncology. We emphasize that membrane–ion dysregulation is included in the proposed framework not because it is currently supported by evidence comparable to that for redox imbalance or mitochondrial priming, but because accumulating findings from apoptosis biology and cancer-cell physiology suggest that it represents an important and underexplored area for future natural-product research. Accordingly, this section should be interpreted as hypothesis-generating rather than as a summary of an established mechanism of phytochemical-induced selective apoptosis. Most phytochemical studies interpret cell death through ROS accumulation, mitochondrial dysfunction, BCL-2-family remodeling, and caspase activation, whereas comparatively few directly investigate whether ion gradients, water movement, osmotic pressure, membrane potential, or cell-volume regulation influence apoptotic sensitivity. Nevertheless, apoptosis is not solely a biochemical process but also involves biophysical changes, including K^+^ efflux, Cl^−^ movement, Ca^2+^ signaling, aquaporin-dependent water transport, membrane tension, VRAC activity, and AVD, whose causal contribution to phytochemical-induced selective apoptosis remains to be established.

In established apoptosis biology, ion flux and AVD can occur early and contribute to caspase activation, mitochondrial dysfunction, and irreversible cell shrinkage. In cancer biology, the same systems are frequently remodeled to sustain proliferation, migration, invasion, metabolic adaptation, survival, and resistance to death. These changes may create dependencies that are not visible when natural products are evaluated only by viability, ROS or mitochondrial readouts. Thus, cancer cells may be selectively sensitive to phytochemicals and other plant-derived compounds not only because they are redox-stressed or mitochondrially primed, but because they depend on altered membrane–ion homeostasis to maintain survival. Membrane–ion biology may therefore represent an underexplored candidate biophysical link between phytochemical-induced stress and apoptotic commitment.

The critical gap is methodological. Natural-product studies rarely test whether K^+^, Cl^−^, Ca^2+^, VRAC/LRRC8, aquaporins, membrane potential, or AVD are causal determinants of response. As a result, membrane-active effects are often interpreted either as nonspecific toxicity or are overlooked entirely. A vulnerability-state approach requires direct measurement of ion flux, cell volume, membrane integrity, mitochondrial coupling and normal-cell sparing, together with rescue experiments using ion-channel inhibitors, osmotic modulation, calcium chelation or genetic perturbation of relevant transporters. Table 6 summarizes how ion–volume modules may cooperate with redox and mitochondrial stress to shape natural-product sensitivity [1,2,3,5,11,20,21,22,36,62,88,89,102,103].

A key starting point is AVD. Cell shrinkage is one of the classical morphological features of apoptosis, but it is not merely a passive consequence of cell dismantling. Loss of K^+^, Cl^−^ and osmotically coupled water can reduce intracellular ionic strength and create a permissive environment for caspase activation, nuclease activity, and apoptosome function. This places ion movement upstream or alongside canonical apoptotic execution, rather than downstream of it. Contemporary reviews of ion-channel-regulated cell death emphasize that volume-sensitive anion channels, acid-sensitive channels, and TRP channels can influence whether cells undergo apoptosis or resist death after stress exposure [104]. Therefore, in natural-product research, early cell shrinkage should be treated as a mechanistic signal that requires measurement, not simply as an image-based marker of apoptosis.

Potassium and chloride fluxes are central to this concept. K^+^ efflux influences membrane potential and intracellular ionic strength, whereas Cl^−^ efflux cooperates with K^+^ loss to drive water efflux and shrinkage. In cancer cells, the same channels that support proliferation and migration may also determine sensitivity to stress-induced death. Recent cancer-focused reviews describe ion channels as contributors to several cancer hallmarks, including proliferation, invasion, and apoptotic resistance, and position them as potential therapeutic targets rather than accessory transport proteins [105]. This is particularly relevant for plant-derived compounds because phytochemicals such as curcumin, quercetin, resveratrol, EGCG, berberine, and capsaicin are frequently reported to alter calcium signaling, membrane potential, or apoptosis, whereas the causal role of K^+^ or Cl^−^ flux is rarely tested directly. A stronger mechanistic interpretation would require showing that ion flux precedes caspase activation and that ionic rescue or channel inhibition attenuates apoptosis.

VRACs provide a particularly attractive but still immature point of connection. VRACs, formed by LRRC8-family proteins, are best known for regulatory volume decrease after swelling, but they also transport organic osmolytes and can participate in stress signaling, drug handling, and cell-death regulation. Recent structural and physiological work has clarified that LRRC8 composition shapes VRAC permeability and substrate selectivity, while cancer-related studies suggest that LRRC8A may have prognostic and functional relevance in different tumor contexts [106]. Importantly, this does not imply that plant-derived compounds generally exert their effects through VRAC. Rather, VRAC should be framed as a plausible candidate mechanism through which membrane-active phytochemicals might interact with osmotic stress, apoptotic volume regulation or drug sensitivity. Claims of VRAC involvement require LRRC8 perturbation, swelling-activated current measurement or volume-recovery assays.

Calcium signaling links the plasma membrane, ER and mitochondria to apoptotic commitment. Transient Ca^2+^ signals can support proliferation and metabolism, whereas sustained Ca^2+^ influx or excessive ER-to-mitochondria transfer can promote mitochondrial depolarization, ROS generation, permeability transition, cytochrome-c release and caspase activation. Mitochondrial calcium handling is increasingly recognized as a determinant of cancer metabolism and therapeutic response, and mitochondrial ion channels are being explored as pharmacological targets in oncology [83]. This axis is highly relevant to plant-derived compounds because many phytochemicals influence calcium-dependent stress responses, although mechanistic specificity remains a major challenge. Calcium elevation alone does not establish a channel-mediated mechanism; source, timing, subcellular localization, and rescue experiments are necessary.

The originality of this vulnerability state lies in its integrative role. Ion fluxes and volume regulation may not act independently from ROS or mitochondrial priming; instead, they may determine whether redox and mitochondrial stress reach the threshold required for irreversible apoptosis. For example, a phytochemical that modestly increases ROS may become more lethal in a cancer cell with fragile K^+^/Cl^−^ homeostasis, defective regulatory volume decrease, or enhanced mitochondrial calcium uptake. Conversely, the same compound may be tolerated if ion-volume adaptation remains intact. This model also helps distinguish apoptosis from necrosis: coordinated K^+^/Cl^−^ loss, AVD, mitochondrial dysfunction and caspase activation suggest regulated apoptosis, whereas early uncontrolled membrane rupture suggests primary toxicity.

Overall, membrane–ion dysregulation should be presented as a research frontier rather than a settled mechanism of phytochemical action. The most rigorous studies should combine time-resolved measurements of cell volume, K^+^, Cl^−^, Ca^2+^, membrane potential, mitochondrial function and caspase activity, together with pharmacological and genetic perturbation of candidate channels. In this framework, ion-channel and cell-volume biology may explain why some cancer cells transition from an adaptive stress response to irreversible apoptosis after natural-product exposure, while others survive or die through non-apoptotic membrane damage.

## 8. Vulnerability State V: Survival-Pathway Addiction

Survival-pathway addiction explains why phytochemical-induced stress may trigger apoptosis in some cancers but remain tolerable in others. Rather than relying on a single oncogenic pathway, cancer cells maintain viability through interconnected adaptive networks, including PI3K–Akt–mTOR, MAPK, NF-κB, STAT3, Wnt–β-catenin, AMPK, autophagy, and the unfolded-protein response. These pathways buffer oxidative, metabolic, mitochondrial, and therapy-induced stress and collectively determine the capacity of cancer cells to withstand injury. Table 7 summarizes the principal survival modules through which plant-derived natural products may weaken this adaptive buffering [1,2,5,22,42,62,79,102,107,108,109,110,111,112,113,114,115].

The PI3K–Akt–mTOR pathway is one of the best-characterized examples of survival-pathway addiction. By regulating metabolism, protein synthesis, autophagy, and anti-apoptotic signaling, this network enables many tumors to tolerate oxidative and mitochondrial stress [116,117]. Likewise, AMPK and MAPK pathways exert context-dependent effects: AMPK may either restore metabolic homeostasis or suppress anabolic growth, whereas ERK, JNK, and p38 signaling can promote proliferation, stress adaptation, or apoptosis depending on activation dynamics and cellular context [118,119,120].

Inflammatory signaling provides another major survival module. NF-κB and STAT3 integrate cytokine signaling, hypoxia, immune evasion, angiogenesis, and anti-apoptotic gene expression, thereby sustaining tumor progression and therapeutic resistance [121,122]. Similarly, Wnt–β-catenin signaling coordinates proliferation, stem-cell maintenance, epithelial–mesenchymal transition, and treatment resistance, making Wnt-dependent tumors particularly susceptible to disruption of β-catenin-driven transcriptional programs [123].

Autophagy and ER stress further determine whether survival-pathway inhibition is tolerated or converted into apoptosis. Autophagy can preserve cellular homeostasis under nutrient deprivation, oxidative stress, or mitochondrial damage, but excessive or dysregulated autophagy may cooperate with cell death. Likewise, adaptive ER stress promotes tumor survival under hypoxic and proteotoxic conditions, whereas unresolved stress activates CHOP, JNK, calcium signaling, and BCL-2-family-dependent apoptosis [27,124,125,126].

Many phytochemicals, including curcumin, resveratrol, EGCG, quercetin, berberine, sulforaphane, pterostilbene, ginsenosides, and withaferin A, simultaneously influence multiple survival pathways rather than a single molecular target. Their biological significance therefore lies in coordinated disruption of adaptive signaling networks rather than isolated pathway modulation.

Future studies should define survival-pathway addiction using functional rather than descriptive approaches. Time-resolved pathway analyses, genetic or pharmacological rescue experiments, phenocopy strategies, and comparison with matched non-malignant cells will be essential to distinguish causal weakening of adaptive signaling from secondary pathway changes associated with generalized cytotoxicity and to identify tumors most susceptible to phytochemical-induced apoptosis.

## 9. When Selective Apoptosis Fails: Necrosis, Non-Selective Toxicity, and Hormesis

Selective apoptosis is a useful conceptual framework only when its limitations are clearly recognized. Plant-derived natural products are frequently described as inherently “safe” or “selective,” yet these properties cannot be assumed. Depending on concentration, exposure duration, formulation, extract composition, metabolic conversion, and cellular context, the same phytochemical may induce apoptosis, cytostasis, adaptive stress responses, ferroptosis, necroptosis, pyroptosis, primary necrosis, or nonspecific toxicity. Selective apoptosis should therefore be regarded as an experimentally demonstrated outcome rather than an intrinsic property of plant-derived compounds.

A major source of misinterpretation is inaccurate classification of cell death. The Nomenclature Committee on Cell Death emphasizes that apoptosis, ferroptosis, necroptosis, pyroptosis, and accidental necrosis are mechanistically distinct processes requiring integrated morphological, biochemical, and functional evidence rather than isolated molecular markers [127]. Accordingly, reduced metabolic viability, ROS accumulation, lipid peroxidation, mitochondrial depolarization, Annexin V positivity, or caspase activation should not individually be interpreted as proof of a specific death modality. Table 8 provides a practical framework for interpreting natural-product-induced cellular outcomes and distinguishing apoptosis, ferroptosis, necroptosis, pyroptosis, accidental necrosis (direct membrane lysis), cytostasis, and mixed regulated cell-death phenotypes [19,23,66,127,128]. Classification should integrate temporally ordered molecular events with characteristic morphology, pathway-specific functional validation, and rescue experiments whenever possible, rather than relying on individual biomarkers such as Annexin V positivity or caspase activation alone.

The boundaries between regulated death programs are dynamic rather than absolute. Apoptotic cells that are not efficiently cleared may progress to secondary necrosis, whereas apoptosis, ferroptosis, necroptosis, and pyroptosis frequently share upstream stress responses and exhibit extensive molecular crosstalk, making pathway-specific validation essential for accurate death-mode classification [128]. Likewise, lipid peroxidation should not automatically be interpreted as apoptosis because ferroptosis is defined by iron-dependent phospholipid peroxidation together with impairment of GPX4/SLC7A11-mediated antioxidant defense and characteristic rescue by ferroptosis-specific inhibitors [19,66]. Similarly, high concentrations of a phytochemical may shift the dominant cellular response from regulated apoptosis toward a mixed regulated cell-death phenotype or nonspecific membrane injury. Consequently, overlapping molecular markers should be interpreted cautiously and within the context of pathway-specific validation.

Dose-dependent nonspecific toxicity represents another important limitation. Membrane-active phytochemicals, particularly saponins and related amphipathic compounds, interact with cholesterol-containing membranes, potentially enhancing cancer-cell stress but also causing direct membrane disruption or hemolysis at higher concentrations [11]. Ginsenosides illustrate the balance between promising anticancer mechanisms and limitations imposed by toxicity, formulation, and pharmacokinetics [87]. Apparent cytotoxicity should therefore not be interpreted as evidence of selective apoptosis without excluding primary membrane injury using appropriate membrane-integrity and normal-cell assays.

Hormesis provides an additional explanation for divergent biological responses. Many phytochemicals exhibit biphasic dose–response relationships in which low concentrations activate adaptive stress responses, antioxidant defenses, or cytoprotective autophagy, whereas higher concentrations promote oxidative injury and regulated cell death [150]. This phenomenon is particularly relevant for redox-active polyphenols. Flavonoids, catechins, and curcuminoids may function as antioxidants, anti-inflammatory agents, or pro-oxidant apoptosis inducers depending on concentration, metal availability, basal ROS, and antioxidant-buffering capacity [2,3,115]. Antioxidant-to-pro-oxidant switching should therefore be interpreted as a context-dependent biological response rather than a universal anticancer mechanism.

Apparent selectivity may also arise from technical rather than biological factors. Variability in botanical source material, extraction procedures, chemical composition, impurities, assay interference, compound instability, and limited pharmacological exposure can all generate misleading evidence of anticancer activity [7,12,13]. Curcumin illustrates this challenge: despite extensive mechanistic investigation, concerns regarding chemical stability, bioavailability, assay interference, and formulation dependence continue to complicate interpretation of its biological effects [5,8,63,151]. Likewise, modulation of drug transporters or activity observed at supraphysiological concentrations does not necessarily predict therapeutic safety, and evidence from dietary carotenoids demonstrates that high-dose supplementation may not reproduce the effects of whole-food exposure and may even be harmful in selected populations [49,50].

Selective apoptosis should therefore be regarded as a testable biological outcome rather than an assumed characteristic of plant-derived natural products. Apparent selectivity may instead reflect misclassified cell death, excessive exposure, adaptive hormesis, technical artifacts, or nonspecific toxicity. Applying the classification framework summarized in Table 8 provides a practical basis for distinguishing these possibilities before attributing selective apoptosis to a plant-derived compound or botanical preparation.

## 10. Normal-Cell Sparing and Immune Compatibility

Normal-cell sparing is the defining translational criterion of selective apoptosis. Cytotoxicity in cancer cells alone does not establish therapeutic value; rather, selective apoptosis should be interpreted as a comparative biological property requiring the preferential induction of apoptosis in malignant cells while preserving the viability and function of biologically relevant matched non-malignant cells. Accordingly, normal-cell sparing should be considered a primary biological endpoint rather than a secondary safety assessment. Demonstration that one cancer cell line is more sensitive than an unrelated normal cell type does not, by itself, establish a clinically meaningful therapeutic window. Instead, evidence of selectivity should be based on biologically appropriate matched normal comparators evaluated under comparable experimental conditions. By contrast, differential sensitivity among cancer cell lines or molecular subtypes primarily reflects differences in cancer-cell vulnerability architecture and identifies distinct vulnerability states rather than demonstrating selective apoptosis itself.

The selectivity index provides a practical starting point by comparing cytotoxicity in normal and malignant cells. Increasingly, phytochemical studies report selectivity indices using non-cancerous comparator cells, improving discrimination between preferential anticancer activity and general cytotoxicity [9,10,152]. However, the value of the selectivity index depends on the comparator model. A favorable index obtained with a single immortalized “normal” cell line cannot predict safety across immune, epithelial, vascular, hepatic, or hematologic tissues. Selection of normal-cell models should therefore reflect the expected route of exposure, tissue distribution, and mechanism of action. Whenever feasible, normal comparators should be tissue-matched to the corresponding malignancy and include non-transformed cells with physiological characteristics relevant to the intended clinical application. Interpretation should also consider whether comparator cells are proliferating or quiescent, because differences in cell-cycle status, metabolic activity, and baseline stress responses may substantially influence apparent drug sensitivity independently of true therapeutic selectivity. Likewise, transformed and non-transformed cells often differ in proliferation kinetics; therefore, comparisons based solely on viability endpoints should be interpreted cautiously and, where possible, supported by growth-rate–adjusted analyses or complementary functional assays. Furthermore, selectivity indices should be interpreted together with absolute effect size, concentration–response relationships, exposure duration, and confidence intervals rather than statistical significance alone, since statistically significant differences may not necessarily represent clinically meaningful therapeutic windows.

Hemolysis assays remain particularly useful for membrane-active compounds, including saponins, glycosides, amphipathic phytochemicals, and nanoformulations, because they readily detect erythrocyte membrane disruption [11]. However, erythrocytes lack nuclei, mitochondria, and many stress-response pathways; consequently, minimal hemolysis does not establish safety in nucleated cells. Conversely, hemolysis at high concentrations may simply indicate nonspecific membrane toxicity. Studies using thymocytes have further demonstrated that compounds such as pinocembrin, ecdysterone, glycyrrhetinic acid derivatives, and gossypol can produce proliferative, volume-regulatory, or apoptotic effects that are not detected by erythrocyte assays alone [153,154,155,156,157].

Immune compatibility represents an equally important component of cancer selectivity because preservation of antitumor immune function is essential for successful clinical translation. Because many phytochemicals modulate redox signaling, NRF2, NF-κB, STAT3, autophagy, or multidrug-resistance pathways, they may influence immune-cell function as well as tumor-cell survival. Peripheral blood mononuclear cells (PBMCs), lymphocytes, and thymocytes provide valuable models for assessing immune-cell viability, proliferation, and inflammatory responses. This distinction is biologically important because transient activation of cytoprotective pathways in normal immune cells may preserve tissue homeostasis, whereas persistent activation of the same pathways within tumors can promote survival and therapeutic resistance. Likewise, modulation of drug transporters may alter drug exposure in normal tissues and contribute to drug–drug interactions [89].

Normal epithelial cells, fibroblasts, hepatocytes, and endothelial cells provide complementary information regarding tissue-specific tolerance. Fibroblasts and epithelial cells are relevant for compounds intended for oral, gastrointestinal, dermatologic, or mucosal administration, hepatocytes for metabolism-related toxicity, and endothelial cells for vascular effects, angiogenesis, and drug-delivery strategies. Patient-derived tumor organoids paired with matched normal organoids further improve translational relevance by capturing tissue architecture, cellular heterogeneity, and drug penetration more accurately than conventional two-dimensional cell cultures [158,159,160]. Such matched patient-derived systems currently represent one of the most physiologically relevant experimental approaches for evaluating whether preferential cancer-cell sensitivity is maintained after accounting for tissue-specific biology and microenvironmental influences.

Overall, normal-cell sparing extends beyond the absence of cytotoxicity. The most promising plant-derived natural products are those that preferentially lower the apoptotic threshold in malignant cells while preserving the viability and function of normal structural and immune cells. A convincing claim of cancer selectivity therefore requires concordant evidence from biologically relevant tissue-matched normal comparators, appropriate assessment of proliferating and quiescent cell populations where applicable, quantitative evaluation of selectivity indices together with biological effect size, and confirmation in physiologically relevant models such as matched organoids or primary cells. Demonstrating this differential response is essential for distinguishing biologically meaningful selective apoptosis from nonspecific cytotoxicity and for establishing a clinically relevant rather than merely statistically significant therapeutic window.

## 11. Methodological Roadmap: Studying Selective Apoptosis Induced by Plant-Derived Compounds

**Figure 3:**
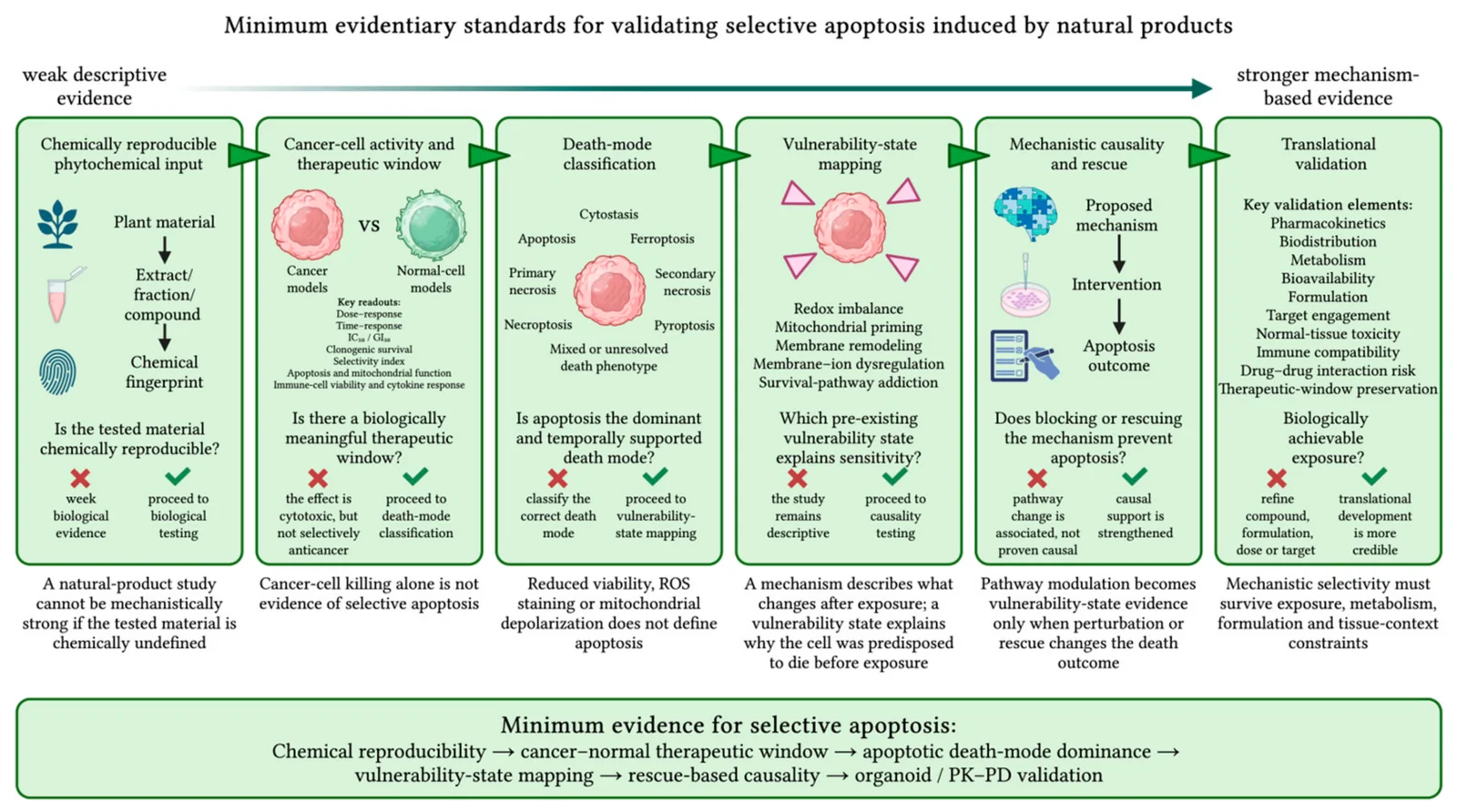
Minimum evidentiary standards for validating selective apoptosis induced by plant-derived compounds. Created in BioRender. Asanova, A. (2026) https://BioRender.com/ylymu25 (accessed on 26 July 2026).

A major limitation of natural-product oncology is the tendency to infer selective apoptosis directly from reduced cancer-cell viability. This evidence alone is insufficient. A compound or extract should be considered selectively pro-apoptotic only when its chemical identity is reproducible, preferential activity toward cancer cells is demonstrated, the dominant mode of cell death is established, the proposed vulnerability state is functionally validated, and translational relevance is supported. Figure 3 summarizes the recommended methodological framework.

1. Phytochemical standardization. Biological interpretation begins with reproducible test material. Studies of extracts and fractions should report botanical authentication, voucher information, plant material and origin, extraction conditions, yield, storage, stability, and reproducible chemical fingerprints using appropriate chromatographic, mass-spectrometric, or spectroscopic methods [7,12,13,14,15]. Without adequate chemical characterization, biological effects cannot be reliably attributed to a specific extract, fraction, or compound.
2. Cytotoxicity and selectivity testing. Dose- and time-response studies should be performed in multiple cancer cell lines together with biologically relevant normal-cell comparators under matched exposure conditions. IC_50_ or 50% growth inhibition (GI_50_) values should be supported by orthogonal viability assays, and the selectivity index should be reported whenever possible [1,6,9,10,152]. Because selectivity depends on the comparator model, evaluation should extend beyond a single immortalized normal cell line to include appropriate structural, immune, or organ-specific cells according to the intended application. Studies should report the rationale for normal-cell selection, document whether comparator cells are proliferating or quiescent, and discuss potential differences in baseline growth kinetics when interpreting selectivity indices.
3. Death-mode classification. Natural-product-induced loss of viability should not be assigned to apoptosis or another regulated death program on the basis of a single biochemical or metabolic endpoint. ROS accumulation, lipid peroxidation, mitochondrial depolarization, phosphatidylserine exposure, caspase activation, and plasma-membrane permeabilization may occur across several death modalities or during late-stage cellular collapse. Classification should therefore combine time-resolved morphology, pathway-associated molecular markers, membrane-integrity measurements, and functional rescue or genetic perturbation. Classification of regulated cell-death modalities should follow the decision framework summarized in Table 8 [19,23,66,127,128].
4. Functional validation of vulnerability states. Proposed vulnerability states should be tested directly rather than inferred from downstream apoptosis. Redox imbalance should be supported by measurements of ROS, glutathione homeostasis, NRF2 activity, lipid peroxidation, and antioxidant rescue [2,3,5,18,36,63]. Mitochondrial priming should be demonstrated through BCL-2-family remodeling, BAX/BAK activation, MOMP, cytochrome c release, and caspase activation in the appropriate temporal sequence [2,22,23,127]. Membrane–ion dysregulation requires direct assessment of membrane integrity, cell-volume regulation, AVD, regulatory volume decrease, ion fluxes, VRAC/LRRC8 activity, and channel-blocker rescue rather than indirect inference from apoptosis alone [11,20,21,161]. Experimental approaches based on thymocyte and erythrocyte models—including hypoosmotic stress, regulatory volume decrease assays, hemolysis, ion-channel analysis, and volume-sensitive anion-channel inhibition—provide practical methods for evaluating membrane integrity and ion-volume regulation [162,163,164,165]. Survival-pathway addiction should be validated by linking alterations in PI3K–Akt–mTOR, AMPK, MAPK, NF-κB, STAT3, Wnt–β-catenin, ER stress, autophagy, or cell-cycle signaling to apoptosis using temporal and rescue experiments [42,103,108,109,114,115].
5. Mechanistic and translational validation. Because phytochemicals frequently influence multiple pathways, pathway modulation alone does not establish causality. Time-course analyses, pharmacological inhibitors, rescue experiments, genetic gain- and loss-of-function approaches, and target-engagement studies provide stronger evidence for mechanism [7,8,12,89]. Comparisons among crude extracts, active fractions, and purified compounds can further distinguish genuine bioactivity from effects attributable to complex mixtures or assay artifacts. Finally, findings from two-dimensional cell cultures should be validated in more physiologically relevant systems, including three-dimensional spheroids, patient-derived tumor and matched normal organoids, primary tumor cultures, stromal or immune co-cultures, and appropriate animal models [158,159,160,166,167]. These models are essential for evaluating pharmacological exposure, metabolism, bioavailability, and safety before therapeutic conclusions are drawn. From a translational perspective, pharmacokinetic and pharmacodynamic plausibility should be evaluated using complementary criteria rather than in vitro potency alone. In particular, the concentrations producing biological effects in vitro should be interpreted alongside unbound plasma exposure, target-tissue or intratumoral exposure where available, exposure to active metabolites, plasma protein binding, achievable duration of pharmacologically active exposure, formulation-dependent drug delivery, and the maximum tolerated dose or clinically achievable exposure. These comparisons help determine whether experimentally active concentrations are pharmacologically attainable and sustained in vivo. Studies should additionally demonstrate target engagement or pathway modulation at clinically relevant exposures, confirm that the proposed mechanism remains operative in physiologically relevant experimental models, and establish consistency between pharmacokinetic exposure, pharmacodynamic responses, and therapeutic outcomes. Collectively, these criteria provide a practical framework for assessing the translational potential of investigational phytochemicals and distinguishing pharmacologically plausible candidates from compounds with activity limited to experimental conditions [168,169,170,171,172].
6. Analytical validation of natural-product activity. Analytical validation is an essential complement to biological validation because apparent pharmacological activity may arise from chemical or assay-related artifacts rather than genuine target modulation (Table 9). Natural products and phytochemicals present additional challenges owing to their structural complexity, variable composition, chemical instability, and susceptibility to assay interference. Consequently, mechanistic conclusions should not rely solely on observed cytotoxicity or pathway modulation but should first exclude common sources of experimental bias [173]. Candidate compounds should therefore undergo systematic analytical quality assessment before mechanistic interpretation. Recommended validation includes confirmation of chemical identity and purity, assessment of batch-to-batch consistency for botanical materials, evaluation of compound stability under experimental conditions, exclusion of colloidal aggregation, redox cycling, nonspecific covalent reactivity, and fluorescence or absorbance interference with assay readouts. Because metabolism may generate active or inactive products, metabolite formation should also be considered whenever pharmacokinetic data are available. Orthogonal analytical methods and chemically independent biological assays should be used whenever feasible to confirm that the observed activity reflects genuine pharmacological effects rather than analytical artifacts [174,175]. These analytical quality-control measures should be considered complementary to the biological validation framework proposed in this review. Together with phytochemical standardization, selective cytotoxicity testing, mechanistic validation, and translational pharmacology, they provide a practical strategy for distinguishing reproducible therapeutic candidates from compounds whose apparent activity primarily reflects experimental limitations [173,174,175,176].

Together, these sequential steps establish whether the tested material is chemically reproducible, analytically robust, selectively cytotoxic, genuinely pro-apoptotic, causally linked to a defined cancer-cell vulnerability state, and pharmacologically plausible under clinically relevant exposure conditions. By integrating phytochemical standardization, analytical validation, mechanistic interrogation, and translational pharmacology, this framework provides a practical roadmap for distinguishing reproducible therapeutic candidates from compounds whose apparent activity primarily reflects experimental limitations. Figure 3 summarizes these methodological principles and their application to mechanism-based phytopharmacology.

## 12. Integrative Model: How Vulnerability States Interact

### 12.1. Selective Apoptosis as a Systems-Level Threshold Event

The central concept of this review is that selective apoptosis induced by plant-derived compounds is a systems-level threshold event rather than the consequence of a single molecular target. Although phytochemical studies often describe ROS generation, mitochondrial dysfunction, membrane perturbation, or survival-pathway inhibition as independent mechanisms, these processes function as interconnected components of a unified stress-response network. The biological outcome depends on the pre-existing vulnerability of the target cell and the extent to which adaptive buffering can prevent irreversible apoptotic commitment. Thus, the critical question is not simply whether a compound induces stress, but whether it selectively drives malignant cells beyond the apoptotic threshold while sparing matched normal cells.

Mitochondria represent the principal integration point within this framework. Redox imbalance, calcium dysregulation, ER stress, metabolic perturbation, DNA damage, and withdrawal of survival signaling all converge on MOMP, where cumulative stress is converted into apoptotic commitment [23]. Likewise, the biological consequences of ROS depend on baseline redox status: cancer cells with elevated oxidative stress rely heavily on glutathione-, thioredoxin-, and NRF2-dependent buffering, whereas cells with greater adaptive reserve may respond to the same phytochemical by activating cytoprotective rather than pro-apoptotic pathways [16]. Autophagy further modulates this threshold by either restoring cellular homeostasis or facilitating mitochondrial dysfunction and apoptosis when adaptive capacity is exceeded [22].

### 12.2. Feed-Forward Loops Between Vulnerability States

The five vulnerability states reinforce one another through feed-forward interactions rather than functioning independently. Redox imbalance promotes mitochondrial dysfunction, whereas dysfunctional mitochondria further amplify ROS production and impair ATP-dependent ion homeostasis. Membrane remodeling alters compound uptake, cholesterol-rich signaling domains, mechanotransduction, and calcium influx, while ion dysregulation influences cell-volume control, intracellular ionic balance, and apoptotic permissiveness. Survival pathways determine whether these stresses remain buffered or progress toward irreversible injury. ROS illustrate this integration particularly well: physiological ROS support signaling, but loss of redox homeostasis promotes oxidative damage and apoptotic commitment [177]. In cancer cells, additional ROS may therefore shift an already stressed system beyond its adaptive capacity [178].

Plant-derived compounds are particularly well suited to reveal these interactions because many simultaneously perturb multiple vulnerability states. Curcumin-related compounds modulate redox balance, mitochondrial function, autophagy, inflammatory signaling, and survival pathways [5]. Plant-derived alkaloids link oxidative stress with mitochondrial apoptosis [36], flavonoids coordinate redox regulation with BCL-2-family proteins and PI3K–Akt, MAPK, and NF-κB signaling [2], and saponins additionally target cholesterol-rich membranes and lipid-raft organization while also carrying a risk of nonspecific membrane toxicity [11]. Together, these observations support the concept that selective apoptosis emerges through coordinated disruption of multiple adaptive networks rather than isolated pathway modulation.

### 12.3. Four Possible Outcomes After Natural-Product Exposure

Within this framework, phytochemical exposure may produce four broad outcomes. First, adaptive survival occurs when stress activates antioxidant defenses, autophagy, mitochondrial quality control, or NRF2-dependent cytoprotection [17,18,179]. Second, cytostasis results from inhibition of proliferation or induction of cell-cycle arrest without dominant apoptotic commitment [3,180]. Third, selective apoptosis occurs when cumulative stress exceeds the apoptotic threshold in malignant cells while remaining tolerable in matched normal cells [42,62,115]. Finally, excessive stress may lead to nonspecific toxicity or alternative regulated death pathways, reflecting membrane injury, ferroptosis, or other context-dependent mechanisms rather than selective apoptosis [7,8,11,19].

### 12.4. Normal-Cell Sparing as the Decisive Filter

Normal-cell sparing remains the decisive filter separating selective vulnerability exploitation from general toxicity. The same phytochemical-induced stress may exceed the apoptotic threshold in cancer cells while remaining tolerable in normal cells with greater antioxidant capacity, mitochondrial reserve, membrane integrity, and metabolic adaptability. Consequently, preferential activity in biologically relevant non-malignant models remains essential for establishing therapeutic selectivity [9,10,11,160].

### 12.5. Experimental Implication: Define the Vulnerability Configuration

Experimentally, the framework shifts attention from asking whether a phytochemical kills a particular cancer cell line to defining the vulnerability configuration that determines sensitivity. Future studies should compare susceptible, resistant, and non-malignant models to identify which vulnerability states are already engaged before treatment and which are required for apoptotic commitment. The experimental strategies needed to establish these causal relationships are outlined in Section 11 [1,6,12,159].

Thus, biological outcome is determined not by compound identity alone, but by the match between phytochemical-induced stress and the pre-existing vulnerability architecture of the target cell.

### 12.6. Illustrative Application of the Vulnerability-State Framework

The practical value of the vulnerability-state framework can be illustrated by applying a common sequence of criteria to representative plant-derived agents: chemical identity, pre-existing cancer-cell vulnerability, induced stress, buffering failure, causal validation, cancer-versus-normal-cell selectivity, and pharmacokinetic or in vivo support. These examples demonstrate how the framework distinguishes mechanistically and translationally mature evidence from findings that remain primarily preclinical.

β-Lapachone exemplifies a biomarker-defined redox vulnerability. This chemically defined naphthoquinone is bioactivated by NQO1, generating a futile redox cycle that produces ROS, DNA damage, PARP1 hyperactivation, and rapid NAD^+^/ATP depletion. The critical vulnerability is high NQO1 activity combined with insufficient antioxidant buffering rather than oxidative stress alone. Genetic or pharmacological inhibition of NQO1 markedly attenuates cytotoxicity, providing direct causal evidence. In hepatocellular carcinoma, the NQO1-to-catalase ratio distinguishes sensitive tumors from normal tissues, while xenograft and metabolic studies support in vivo efficacy. Nevertheless, therapeutic activity remains dependent on tumor NQO1 expression, antioxidant capacity, formulation, and achievable exposure [181,182].

Piperlongumine illustrates transformation-associated dependence on antioxidant buffering but with greater translational uncertainty. This purified alkaloid preferentially kills diverse cancer and oncogenically transformed cells while showing lower toxicity in several primary normal-cell models. It induces ROS accumulation, disrupts glutathione homeostasis, activates stress pathways including JNK, and triggers compensatory NRF2–HO-1 signaling; antioxidant rescue and JNK inhibition support a causal role for oxidative stress. Although xenograft studies demonstrate antitumor activity, electrophilic reactivity, possible nonspecific protein modification, and limited pharmacokinetic evidence indicate that piperlongumine remains a promising but incompletely validated vulnerability-directed agent [183,184,185].

Parthenolide and its orally bioavailable analog dimethylamino-parthenolide (DMAPT) illustrate selective disruption of leukemia-cell survival buffering. Parthenolide preferentially targets acute myeloid leukemia and blast-crisis chronic myeloid leukemia stem/progenitor cells over normal hematopoietic counterparts by combining ROS induction with NF-κB inhibition and apoptosis. Antioxidant rescue and pathway-directed studies support mechanistic causality. Development of DMAPT overcame poor bioavailability while preserving selective antileukemic activity and demonstrating in vivo efficacy, thereby linking chemical optimization with improved translational potential. Remaining uncertainties include electrophilic promiscuity, multiple molecular targets, and limited clinical validation [186,187].

Together, these examples demonstrate that the framework identifies both supporting evidence and remaining uncertainties. β-Lapachone represents a biomarker-driven model with strong mechanistic and pharmacological support, piperlongumine illustrates cancer-associated oxidative vulnerability but incomplete translational validation, and parthenolide/DMAPT shows how medicinal chemistry can improve the clinical potential of a phytochemical while preserving selective activity. Rather than implying equivalence among plant-derived agents, the framework grades the convergence of chemical quality, vulnerability mapping, causal validation, normal-cell selectivity, and pharmacological plausibility.

At the same time, the proposed framework has several important limitations. Vulnerability states should not be regarded as fixed biological properties but as dynamic features that evolve during tumor progression, metastatic dissemination, and therapeutic selection. Consequently, the relative importance of individual vulnerability states is expected to vary across tumor types, molecular subtypes, and treatment-resistant populations according to their genetic, epigenetic, metabolic, and microenvironmental context. Moreover, the framework is intended as an integrative conceptual model for interpreting existing evidence rather than as a predictive algorithm capable of assigning vulnerability states to individual patients. Its future clinical implementation will require robust biomarkers, standardized approaches for vulnerability-state assessment, longitudinal validation during disease evolution, and prospective evaluation in clinically relevant models. Finally, several proposed interactions—particularly those involving membrane–ion biology—remain supported predominantly by preclinical evidence and require further mechanistic and translational validation. Accordingly, the vulnerability-state framework should be viewed as a hypothesis-generating tool that guides mechanistic investigation, biomarker development, and vulnerability-directed phytopharmacology rather than as a definitive model of cancer-cell behavior.

## 13. Future Directions

The next stage of natural-product oncology should move beyond cataloging bioactive compounds toward identifying the vulnerability states that determine selective apoptosis. Rather than asking which phytochemicals kill cancer cells, future studies should define the baseline biological features that distinguish sensitive, resistant, and normal cells. These include redox imbalance, mitochondrial priming, membrane remodeling, ion-channel dependence, survival-pathway addiction, and the adaptive reserve that determines proximity to apoptotic commitment. Integrating these features into predictive vulnerability signatures may improve patient stratification and reveal biomarkers of phytochemical responsiveness.

A major research priority is to establish the causal hierarchy among vulnerability states. Although redox stress, mitochondrial dysfunction, membrane remodeling, ion dysregulation, and survival-pathway inhibition frequently occur together, their temporal and mechanistic relationships remain poorly defined. Future studies should determine which vulnerabilities initiate apoptosis, which amplify the response through feed-forward interactions, and which represent secondary consequences of irreversible cell death. Perturbation and rescue strategies will be essential for distinguishing causal drivers from downstream biomarkers.

Membrane–ion biology represents one of the most underexplored areas in natural-product research. While most studies focus on oxidative stress and mitochondrial signaling, comparatively little is known about how LRRC8/VRAC channels, aquaporins, calcium, potassium, and chloride fluxes, or AVD contribute to selective apoptosis. Clarifying whether membrane–ion dysregulation acts as an initiating vulnerability or merely accompanies cell death will substantially refine the proposed framework and improve discrimination between regulated apoptosis and nonspecific membrane injury.

Translation will also require models that better capture patient biology. Patient-derived tumor organoids, matched normal organoids, ex vivo tumor cells, and pharmacokinetic/pharmacodynamic-informed experimental systems provide opportunities to evaluate whether vulnerability-state configurations predict therapeutic response in clinically relevant settings. Such approaches may facilitate identification of biomarker-defined patient subgroups most likely to benefit from phytochemical-based interventions.

Another important direction is the rational integration of plant-derived compounds with established anticancer therapies. Because many phytochemicals modulate multiple adaptive stress networks simultaneously, they may be particularly effective when combined with chemotherapy, targeted therapy, BH3 mimetics, redox-directed agents, pathway inhibitors, radiotherapy, or immunotherapy. Future combination strategies should focus on overcoming adaptive resistance while preserving normal-cell and immune-cell function.

Ultimately, successful clinical translation will depend on demonstrating activity at pharmacologically achievable exposures, identifying robust pharmacodynamic biomarkers, optimizing formulations, and establishing safety together with immune compatibility. The next generation of natural-product oncology should therefore move from compound cataloging toward vulnerability mapping. Linking chemically defined phytochemical stress to predictive cancer-cell states, causal validation, pharmacologically plausible exposure, and preservation of normal and immune function has the potential to transform descriptive cytotoxicity into mechanism-informed phytopharmacology.

## 14. Conclusions

Plant-derived natural products remain a major source of anticancer chemical diversity, but their translational value depends on moving beyond compound catalogs and descriptive pathway reporting toward understanding the biological contexts that determine selective activity. In this review, we propose a vulnerability-state framework in which selective apoptosis arises when stress induced by chemically characterized plant-derived compounds intersects with pre-existing cancer-cell vulnerabilities, exceeds adaptive buffering capacity, and remains below the injury threshold of relevant normal and immune cells. This perspective shifts the central question from whether a plant-derived compound kills cancer cells to which biological state renders a cancer cell selectively susceptible to phytochemical-induced apoptosis.

An important implication is that molecular mechanisms and vulnerability states should not be conflated. ROS generation, mitochondrial depolarization, BCL-2-family remodeling, caspase activation, survival-pathway modulation, and membrane disruption describe biological responses following phytochemical exposure; they become evidence of vulnerability only when they explain differential sensitivity between malignant and non-malignant cells or between sensitive and resistant cancer states. Within this framework, membrane–ion dysregulation represents a particularly promising area for future investigation, complementing established concepts such as redox imbalance, mitochondrial priming, and survival-pathway dependence.

The proposed framework should be viewed as a conceptual integration rather than a new biological theory. Its contribution is not the introduction of previously unknown mechanisms but the organization of established concepts into a unified, phytopharmacology-oriented model that facilitates the interpretation of mechanistic studies, comparison across phytochemical classes, and generation of testable hypotheses regarding selective apoptosis. By emphasizing biological vulnerability states rather than individual compounds alone, this framework may contribute a complementary perspective for guiding future mechanistic, translational, and natural-product-based anticancer research.

## Figures and Tables

**Table 1:** Relationship between the proposed vulnerability-state framework and established concepts in cancer biology.

Established Concept	Established Role in Cancer Biology	Relationship to the Proposed Vulnerability-State Framework
Mitochondrial priming	Determines how close a cell is to the apoptotic threshold.	Integrated as one component of the pre-existing vulnerability architecture that influences susceptibility to phytochemical-induced apoptosis.
Redox adaptation	Maintains oxidative homeostasis through antioxidant-buffering systems.	Forms the basis of the redox imbalance vulnerability state, where limited buffering capacity permits selective oxidative stress.
Oncogene addiction/survival-pathway dependence	Dependence on oncogenic signaling for proliferation and survival.	Interpreted as survival-pathway addiction that renders cancer cells selectively sensitive to phytochemical-mediated pathway disruption.
Stress-buffer dependence	Reliance on adaptive stress-response systems to maintain viability.	Incorporated through the concept of adaptive buffering capacity, whose failure permits apoptotic commitment.
Therapeutic window/cancer-selective vulnerability	Differential sensitivity between malignant and normal cells.	Expanded by explicitly incorporating normal-cell sparing as a defining requirement for selective apoptosis.
Synthetic lethality	Cell death resulting from specific combinations of genetic defects and targeted perturbations.	Complementary rather than equivalent. The proposed framework focuses on phytochemical-induced stress interacting with pre-existing biological vulnerability states rather than defined genetic interactions.

Note: The concepts summarized in the first two columns are well established in cancer biology and are discussed throughout this Review. The proposed framework does not introduce new biological mechanisms but integrates these complementary concepts into a unified phytopharmacology-oriented model for interpreting plant-derived natural-product-induced selective apoptosis.

**Table 2:** Conceptual interpretation of major phytochemical classes within the proposed vulnerability-state framework of selective apoptosis.

Phytochemical Class	Conceptual Role in the Framework	Principal Vulnerability Axis	Evidence Level	Major Translational Considerations
Alkaloids	Clinically validated alkaloid-derived drugs (e.g., vinca alkaloids, camptothecin derivatives) validate mitotic-spindle and DNA-topology stress, whereas investigational alkaloids illustrate ROS–mitochondria–apoptosis interactions in preclinical models.	Mitotic stress; DNA-topology stress; mitochondrial/ROS vulnerability	Clinical (approved drugs); preclinical (other alkaloids)	Strong clinical precedent exists for approved alkaloids. Berberine, piperine, matrine, and sanguinarine remain investigational because of limitations in toxicity, solubility, delivery, off-target effects, and therapeutic window [34,35,36].
Flavonoids	Mechanistic exemplars of context-dependent redox and inflammatory-survival signaling rather than a uniform anticancer class.	Redox buffering; inflammatory-survival signaling; EMT/metastatic plasticity	Predominantly preclinical	Translation is limited by poor bioavailability, rapid metabolism, microbiome-dependent transformation, and frequent use of supraphysiological in vitro concentrations [2,3,37].
Phenolic acids and simple phenolics	Illustrate prevention-oriented redox and inflammatory modulation rather than direct systemic cytotoxic therapy.	Redox-state vulnerability; inflammatory microenvironment; mitochondrial priming	Preclinical; strongest for prevention	Evidence favors chemoprevention and microenvironment modulation rather than systemic anticancer therapy. Activity may depend on conjugated or microbiota-derived metabolites [38,39,40].
Stilbenes	Demonstrate that extensive pathway modulation does not necessarily indicate selective apoptosis without biologically achievable exposure.	Survival-pathway dependence; metabolic stress; mitochondrial vulnerability	Predominantly preclinical	Clinical translation remains limited by rapid metabolism, low systemic exposure, and uncertain relevance of high in vitro concentrations [41,42,43].
Curcuminoids	Paradigm example highlighting the need to distinguish pathway modulation from validated selective apoptosis.	Adaptive stress; inflammatory-survival signaling; therapy resistance; selected redox/ferroptosis-associated vulnerability	Extensive preclinical evidence	Interpretation requires caution because of poor solubility, chemical instability, assay interference, formulation dependence, and inconsistent clinical translation [5,8,44].
Terpenoids and terpenes	Taxanes validate clinically actionable mitotic stress, whereas other terpenoids should be interpreted in a compound-specific manner.	Mitotic stress (taxanes); mitochondrial stress; lipid peroxidation/ferroptosis susceptibility; inflammatory-survival signaling	Clinical (taxanes); preclinical (most non-taxane terpenoids)	Translational evidence is strong for taxanes but remains limited for many other terpenoids because of delivery, pharmacokinetic, toxicity, and target-engagement challenges [34,45,46,47].
Saponins	Illustrate membrane–cholesterol vulnerability while emphasizing the need to distinguish selective membrane perturbation from nonspecific cytotoxicity.	Membrane–cholesterol stress; lipid-raft signaling; calcium/ion-homeostasis vulnerability	Mainly cell-line and limited in vivo evidence	Translation requires careful safety evaluation because membrane-disruptive activity may also cause hemolysis and systemic toxicity [24,48].
Carotenoids	Context-dependent redox modulators rather than direct anticancer cytotoxins.	Redox-threshold stress; mitochondrial ROS vulnerability; lipid-membrane oxidative stress	Clinical prevention; limited oncology evidence	Evidence is strongest for diet and cancer prevention. High-dose isolated supplementation may not reproduce food-matrix effects and may be harmful in selected high-risk populations [49,50,51].
Tannins and hydrolysable/condensed polyphenols	Illustrate metabolite-mediated and local gastrointestinal vulnerability rather than systemic activity of parent compounds.	Gastrointestinal redox-inflammatory stress; microbiome–metabolite vulnerability	Preclinical; prevention-oriented	Biological activity is frequently mediated by gut-derived metabolites rather than the parent compounds. Direct clinical oncology evidence remains limited [52,53,54].
Coumarins	Medicinal-chemistry scaffolds rather than a uniform dietary anticancer class.	Cell-cycle stress; redox stress; drug-resistance vulnerability; pH/metabolic adaptation (selected derivatives)	Predominantly preclinical	Natural coumarins, furanocoumarins, and optimized derivatives should be interpreted separately because they differ substantially in potency, metabolism, and safety [55,56,57].
Glucosinolates and isothiocyanates	Exemplify adaptive-redox biology and the context-dependent role of NRF2 signaling.	Detoxification/adaptive-redox stress; epigenetic stress; cancer stem-cell vulnerability	Preclinical with supportive clinical prevention evidence	NRF2 activation may protect normal tissues during prevention but promote therapy resistance in established NRF2-dependent cancers [17,18,58,59].
Phytosterols and plant steroids	Distinguish dietary phytosterols from pharmacologically active steroidal lactones such as withanolides.	Membrane-lipid/cholesterol stress; proteotoxic, cytoskeletal, inflammatory, and mitochondrial vulnerability	Heterogeneous; mainly preclinical	Dietary phytosterols and withanolides differ markedly in potency and translational potential. Pharmacokinetic and toxicity validation remain necessary [60,61,62].

Note: This table summarizes the conceptual roles of representative phytochemical classes and other plant-derived agents within the proposed vulnerability-state framework rather than providing a comprehensive inventory of plant-derived natural products. Representative compounds and mechanisms should not be generalized to all members of a phytochemical class. The listed vulnerability axes indicate the predominant biological context for interpretation and are not exclusive mechanisms. Evidence level reflects the highest level of support available for representative compounds within each class and should not be interpreted as applying uniformly to the entire class. The categories summarized in this table represent distinct classes of evidence with different standards of chemical characterization, reproducibility, pharmacokinetic validation, and clinical translation and should not be interpreted interchangeably. Abbreviations: EMT, epithelial–mesenchymal transition; ROS, reactive oxygen species.

**Table 3:** Redox features that may lower the apoptotic threshold after natural-product exposure.

Redox Vulnerability Feature	Biological Rationale	Representative Natural-Product Relevance	Evidence Level	Key Validation Criteria
Basal ROS threshold	Cancer cells with elevated basal ROS are closer to the cytotoxic redox threshold and may undergo apoptosis when oxidative stress exceeds antioxidant capacity.	Frequently reported for curcumin, quercetin, EGCG, resveratrol, berberine, piperine, sulforaphane, withaferin A and related redox-active phytochemicals.	Strong preclinical	Demonstrate increased ROS before apoptosis, confirm antioxidant rescue (e.g., NAC or MitoTEMPO), and show preferential effects in matched non-malignant cells.
Mitochondrial ROS–MOMP–caspase coupling	Mitochondrial ROS may promote mitochondrial outer-membrane permeabilization (MOMP), cytochrome-*c* release and caspase activation in susceptible cancer cells.	Reported for curcumin, resveratrol, berberine, quercetin, EGCG, piperine, withaferin A and selected alkaloids.	Strong preclinical	Demonstrate mitochondrial ROS upstream of MOMP and caspase activation using mitochondrial ROS measurements together with pharmacological or genetic rescue.
NRF2–KEAP1 antioxidant adaptation	NRF2-dependent antioxidant responses may protect normal tissues but also promote survival and therapy resistance in established cancers.	Sulforaphane is the best-characterized example; curcumin, quercetin, resveratrol, berberine and EGCG modulate NRF2 signaling in a context-dependent manner.	Strong mechanistic; context-dependent clinical relevance	Assess NRF2 pathway activation together with gain- or loss-of-function experiments to distinguish cytoprotection from apoptosis sensitization.
Glutathione and thioredoxin buffering	Dependence on glutathione and thioredoxin systems may create a vulnerability when antioxidant buffering is impaired.	Reported for curcumin, quercetin, EGCG, berberine, sulforaphane, isothiocyanates and other electrophilic phytochemicals.	Strong mechanistic	Demonstrate functional depletion of glutathione/thioredoxin buffering, together with rescue by restoration of antioxidant capacity.
Cystine–GSH–GPX4 lipid-peroxide defense	Dependence on SLC7A11–GSH–GPX4 may determine susceptibility to lipid peroxidation, ferroptosis or mixed regulated cell-death phenotype under oxidative stress.	Associated with sulforaphane, curcumin, EGCG, baicalein, resveratrol, artemisinin derivatives, berberine and related electrophilic compounds.	Moderate–strong preclinical	Distinguish apoptosis from ferroptosis using lipid-peroxidation markers together with ferroptosis- and caspase-specific rescue experiments.
NADPH-dependent redox metabolism	Limited NADPH production may reduce glutathione and thioredoxin recycling, increasing vulnerability to oxidative stress.	Curcumin, resveratrol, berberine, EGCG and quercetin have been linked to metabolic reprogramming affecting cellular redox balance.	Emerging/mechanistic inference	Directly quantify NADPH metabolism and demonstrate metabolic rescue before inferring a causal role in apoptosis.
NOX-derived ROS and redox-sensitive survival signaling	NOX-derived ROS may regulate PI3K/Akt, MAPK, NF-κB and STAT3 signaling, influencing survival and oxidative adaptation.	Curcumin, quercetin, EGCG, resveratrol, berberine and sulforaphane have been reported to modulate these pathways, although direct attribution to NOX-derived ROS is often incomplete.	Moderate mechanistic	Identify the ROS source and demonstrate that pathway inhibition depends on NOX-derived ROS rather than secondary oxidative stress.
Hypoxia–HIF–redox adaptation	Hypoxia alters mitochondrial ROS, HIF signaling and antioxidant adaptation, potentially creating context-dependent redox vulnerabilities.	Curcumin, resveratrol, quercetin, EGCG, berberine and sulforaphane have been investigated in hypoxia-related preclinical models.	Moderate preclinical	Validate findings under physiologically relevant hypoxia using advanced models and compare with normoxic controls.

Note: Redox imbalance should be interpreted as a vulnerability state rather than a generic mechanism of cell death. The same natural product may exhibit antioxidant, cytostatic, pro-apoptotic or ferroptosis-associated activity depending on basal ROS, antioxidant reserve, mitochondrial function, NRF2/KEAP1 status, lipid-peroxide buffering, dose and exposure duration. Selective apoptosis is most convincingly supported when redox perturbation precedes mitochondrial commitment and caspase activation, is reduced by appropriate rescue experiments, and occurs preferentially in cancer cells compared with relevant non-malignant controls. Evidence levels summarize the highest level of support currently available for each vulnerability feature and should not be interpreted as applying uniformly to every phytochemical listed. Representative phytochemicals are provided solely as conceptual examples to illustrate the predominant vulnerability state. Individual members of a phytochemical class may differ substantially in biological activity, mechanism, pharmacokinetics, and translational relevance; therefore, these characteristics should not be generalized to the entire class. Abbreviations: EGCG, epigallocatechin gallate; MOMP, mitochondrial outer-membrane permeabilization; MitoTEMPO, mitochondria-targeted superoxide dismutase mimetic antioxidant; NAC, *N*-acetylcysteine; ROS, reactive oxygen species.

**Table 4:** Mitochondrial features that may lower the apoptotic threshold after natural-product exposure.

Mitochondrial Vulnerability Feature	Biological Rationale	Representative Natural-Product Relevance	Evidence Level	Key Validation Criteria
BCL-2-family rheostat	A shift from anti-apoptotic (BCL-2, BCL-xL, MCL-1) to pro-apoptotic (BAX/BAK) signaling lowers the threshold for mitochondrial apoptosis.	Frequently reported for curcumin, quercetin, resveratrol, berberine, EGCG, piperine, withaferin A and related phytochemicals.	Strong preclinical	Demonstrate BCL-2-family remodeling preceding MOMP and caspase activation, supported by genetic or pharmacological rescue.
BH3-only stress signaling	BH3-only proteins integrate upstream stress signals (e.g., p53, ROS, ER stress, JNK) to activate BAX/BAK-mediated apoptosis.	Reported for curcumin, resveratrol, quercetin, berberine, EGCG and withaferin A.	Strong mechanistic	Demonstrate functional involvement of BH3-only proteins using gain-/loss-of-function approaches rather than expression changes alone.
Mitochondrial apoptotic priming	Highly primed cells require only modest additional stress to undergo MOMP. Dynamic BH3 profiling directly measures this vulnerability.	Direct BH3 profiling has rarely been applied to phytochemicals and remains an important research gap.	Emerging/mechanistic inference	Demonstrate baseline and dynamic BH3 profiling together with comparison to non-malignant cells and BH3-mimetic synergy where appropriate.
MOMP and cytochrome-c release	MOMP represents the point of irreversible commitment to intrinsic apoptosis.	Frequently reported following treatment with curcumin, quercetin, resveratrol, berberine, EGCG and related phytochemicals.	Strong preclinical	Demonstrate MOMP preceding cytochrome-*c* release and caspase-9 activation using time-course and rescue experiments.
Mitochondrial membrane potential and bioenergetic reserve	Cells with limited respiratory reserve are more susceptible to oxidative phosphorylation disruption and mitochondrial stress.	Reported for curcumin, resveratrol, berberine, quercetin, EGCG and selected terpenoids.	Strong preclinical	Link bioenergetic dysfunction to apoptotic commitment rather than nonspecific mitochondrial injury using functional rescue.
p53-dependent mitochondrial apoptosis	Functional p53 lowers the apoptotic threshold through induction of BAX, PUMA, NOXA and related mitochondrial death pathways.	Reported for curcumin, resveratrol, quercetin, EGCG and berberine in p53-competent models.	Strong mechanistic	Demonstrate dependence on p53 using TP53-defined models or genetic rescue experiments.
p53-independent mitochondrial apoptosis	p53-deficient cells may undergo intrinsic apoptosis through ROS, ER stress, JNK activation or direct BCL-2-family remodeling.	Reported for curcumin, berberine, quercetin, resveratrol, EGCG, piperine and withaferin A.	Moderate mechanistic	Demonstrate apoptosis in p53-deficient models together with pathway-specific rescue experiments.
MCL-1-dependent anti-apoptotic buffering	Dependence on MCL-1 creates vulnerability when phytochemicals reduce MCL-1 or increase NOXA.	Reported in preclinical studies of curcumin, quercetin, resveratrol, berberine, EGCG and withaferin A.	Moderate–strong mechanistic	Demonstrate that MCL-1 loss is causal using overexpression or pharmacological rescue.
BCL-2/BCL-xL dependence and BH3-mimetic-like sensitization	Phytochemicals may sensitize BCL-2/BCL-xL-dependent tumors without directly acting as BH3 mimetics.	Reported for curcumin, quercetin, resveratrol, berberine and EGCG.	Moderate mechanistic	Demonstrate enhanced sensitivity to BH3 mimetics or disruption of anti-apoptotic buffering while avoiding claims of direct BH3-mimetic activity without binding evidence.
Mitochondrial ROS–apoptosis coupling	Excess mitochondrial ROS may trigger MOMP and intrinsic apoptosis in susceptible cancer cells.	Frequently reported for curcumin, berberine, quercetin, resveratrol, EGCG, piperine and withaferin A.	Strong preclinical	Demonstrate mitochondrial ROS upstream of apoptosis using mitochondrial ROS measurements and antioxidant rescue.
ER stress–mitochondria apoptotic coupling	Persistent ER stress may promote mitochondrial apoptosis through CHOP, JNK, BIM, PUMA, NOXA and calcium transfer.	Reported for curcumin, resveratrol, quercetin, berberine, celastrol and withaferin A.	Moderate–strong mechanistic	Demonstrate functional coupling between ER stress and mitochondrial apoptosis using pathway-specific rescue experiments.
Mitochondrial calcium overload and permeability-transition susceptibility	Excessive ER–mitochondrial calcium transfer may promote mitochondrial depolarization, permeability transition and apoptosis, although severe calcium overload may also induce non-apoptotic death.	Reported for curcumin, resveratrol, quercetin, berberine, withaferin A and selected terpenoids.	Moderate mechanistic	Demonstrate calcium-dependent intrinsic apoptosis using calcium imaging together with mitochondrial and caspase-specific rescue experiments.

Note: Mitochondrial priming should be interpreted as a pre-existing vulnerability state rather than a consequence of mitochondrial damage. Within the proposed framework, mitochondrial priming reflects dependence on anti-apoptotic buffering, limited bioenergetic reserve, or increased pro-apoptotic pressure that lowers the threshold for mitochondrial outer-membrane permeabilization. Selective apoptosis is most convincingly supported when BCL-2-family remodeling precedes MOMP, cytochrome-c release, and caspase-9/-3 activation, is attenuated by appropriate rescue experiments, and occurs preferentially in cancer cells compared with relevant non-malignant controls. Evidence levels summarize the highest level of support currently available for each vulnerability feature and should not be interpreted as applying uniformly to every phytochemical listed. Representative compounds are illustrative rather than exhaustive. Abbreviations: EGCG, epigallocatechin gallate; ER, endoplasmic reticulum; MOMP, mitochondrial outer-membrane permeabilization; PUMA, p53-upregulated modulator of apoptosis; ROS, reactive oxygen species.

**Table 5:** Membrane features that shape natural-product uptake, stress signaling and selective toxicity.

Membrane Vulnerability Feature	Biological Rationale	Representative Natural-Product Relevance	Evidence Level	Key Validation Criteria
Cholesterol-rich membranes and lipid-raft dependence	Cancer cells dependent on cholesterol-rich lipid rafts for receptor and survival signaling may become vulnerable to disruption of membrane organization.	Most strongly associated with saponins, including ginsenosides, diosgenin-derived saponins and triterpenoid saponins.	Moderate–strong preclinical	Demonstrate cholesterol or lipid-raft disruption preceding apoptosis using cholesterol depletion/repletion and functional rescue in comparison with matched non-malignant cells.
Membrane permeability and detergent-like membrane stress	Altered membrane composition may increase susceptibility to amphipathic compounds, although excessive membrane damage may produce nonspecific cytotoxicity rather than regulated apoptosis.	Primarily associated with saponins because of their membrane-active properties.	Strong mechanistic	Distinguish regulated apoptosis from primary membrane lysis using time-course analysis, membrane integrity assays and caspase-dependent rescue.
Lipid-raft survival-signaling platforms	Lipid rafts organize EGFR, HER2, Src, Akt, MAPK and NF-κB signaling, creating potential membrane-associated survival vulnerabilities.	Saponins directly disrupt lipid rafts, whereas curcumin, quercetin, EGCG and resveratrol have been reported to modulate raft-associated signaling.	Moderate mechanistic	Demonstrate raft disruption before signaling inhibition and apoptosis using lipid-raft localization together with cholesterol rescue experiments.
Bilayer fluidity, dipole potential and membrane order	Changes in membrane biophysical properties may influence receptor function, transporter activity and apoptotic susceptibility.	Reported for curcumin, resveratrol, quercetin, EGCG and related polyphenols.	Emerging/mechanistic inference	Demonstrate membrane biophysical changes at biologically relevant concentrations together with functional linkage to apoptosis.
Transporter-rich membranes and multidrug resistance	ABC transporter activity may reduce intracellular phytochemical accumulation, whereas transporter inhibition may restore drug sensitivity.	Curcumin, quercetin, resveratrol, berberine, piperine and EGCG have been investigated as MDR modulators.	Moderate–strong preclinical	Demonstrate altered intracellular exposure together with transporter-specific functional assays and chemosensitivity rescue.
Phytochemical delivery across lipid membranes	Membrane permeability and formulation influence intracellular exposure and apparent biological activity.	Lipid-based formulations have been widely studied for curcumin, quercetin, EGCG, resveratrol and related phytochemicals.	Strong translational	Demonstrate improved intracellular delivery separately from enhanced biological activity, using equivalent intracellular exposure and matched normal-cell comparisons.
Phosphatidylserine exposure during apoptosis	Phosphatidylserine externalization is an early marker of apoptosis but does not indicate selective membrane targeting.	Commonly reported for flavonoids, curcuminoids, alkaloids and saponins.	Established apoptosis marker	Interpret Annexin V together with membrane integrity, caspase activation and time-course analyses rather than as a standalone indicator of selective apoptosis.
Ion-channel and calcium-permeability modulation	Ion channels regulate calcium homeostasis, mitochondrial function and apoptotic signaling, potentially creating membrane-associated vulnerabilities.	Curcumin, resveratrol, quercetin, EGCG, cannabinoids, terpenoids and selected alkaloids have been reported to modulate calcium signaling or ion channels.	Moderate mechanistic	Demonstrate direct ion-channel involvement using electrophysiology or channel-specific pharmacological/genetic modulation rather than calcium measurements alone.
Membrane–cytoskeleton coupling	Membrane organization, receptor clustering and endocytosis depend on cytoskeletal architecture, which may influence sensitivity to membrane-active phytochemicals.	Reported for withaferin A, curcumin, resveratrol, quercetin, saponins and selected terpenoids.	Moderate mechanistic	Demonstrate cytoskeletal remodeling preceding membrane changes and apoptosis using functional rescue experiments.

Note: Membrane remodeling should be interpreted as a potential vulnerability state rather than direct evidence of selective apoptosis. Cancer-cell membranes may differ from normal-cell membranes in cholesterol content, lipid-raft organization, membrane order, transporter activity, ion-channel expression and cytoskeletal coupling, potentially influencing phytochemical uptake and stress responses. However, membrane-active phytochemicals may also cause nonspecific membrane disruption, hemolysis or direct cytotoxicity. Selective apoptosis is most convincingly supported when membrane remodeling precedes apoptotic commitment, is functionally linked to downstream signaling through pharmacological or genetic rescue, and occurs preferentially in cancer cells compared with relevant non-malignant controls. Evidence levels summarize the highest level of support currently available for each vulnerability feature and should not be interpreted as applying uniformly to every phytochemical listed. Representative compounds are illustrative rather than exhaustive. Abbreviations: MDR, multidrug resistance; Src, proto-oncogene tyrosine-protein kinase Src.

**Table 6:** Ion-channel and cell-volume features that may cooperate with natural-product-induced apoptosis.

Ion–Volume Vulnerability Feature	Biological Rationale	Representative Natural-Product Relevance	Evidence Level	Key Validation Criteria
Apoptotic volume decrease (AVD)	Early K^+^, Cl^−^, and water loss can promote cell shrinkage, alter intracellular ionic strength, and facilitate caspase activation in apoptosis-prone cells.	Cell shrinkage is frequently reported after exposure to curcumin, quercetin, EGCG, resveratrol, berberine, piperine, withaferin A, and related phytochemicals, but direct evidence that AVD initiates apoptosis remains uncommon.	Moderate mechanistic	Demonstrate that volume loss precedes mitochondrial and caspase activation, directly measure K^+^/Cl^−^ flux, and show rescue by osmotic or ion-channel manipulation.
Regulatory volume decrease and osmotic-stress tolerance	Cancer cells rely on volume-regulatory mechanisms to withstand swelling, confinement, hypoxia, and migration-associated mechanical stress. Disruption of this adaptive capacity may increase apoptotic sensitivity.	Membrane-active saponins and selected phytochemicals may alter membrane permeability or ion homeostasis, but direct evidence that chemically characterized plant-derived compounds selectively kill cancer cells through impaired regulatory volume decrease remains limited.	Emerging/hypothesis-generating	Use real-time swelling and volume-recovery assays, manipulate candidate channels or cotransporters, and demonstrate selective loss of viability under osmotic stress.
K^+^ efflux and intracellular ionic-strength control	Intracellular K^+^ loss can remove inhibition of caspases and nucleases and thereby facilitate apoptotic execution.	Curcumin, resveratrol, quercetin, EGCG, berberine, capsaicin, and related compounds have been linked to altered K^+^ conductance or membrane potential, although direct causal evidence is limited.	Moderate mechanistic	Directly measure intracellular K^+^ loss and channel activity, then show that high extracellular K^+^ or channel-specific manipulation reduces apoptosis.
Cl^−^ efflux and anion-channel-dependent shrinkage	Cl^−^ efflux cooperates with K^+^ loss to drive osmotic water exit and apoptotic shrinkage.	Direct evidence for phytochemical-induced, Cl^−^-dependent apoptosis is sparse; most studies do not distinguish causal Cl^−^ flux from secondary apoptotic changes.	Emerging/mechanistic inference	Measure intracellular Cl^−^ and anion currents, demonstrate temporal precedence, and confirm functional rescue using ion substitution or channel-specific interventions.
VRAC/LRRC8-dependent volume-sensitive anion currents	VRAC regulates swelling-activated Cl^−^ and osmolyte efflux, volume recovery, and stress responses, and may also influence drug transport.	Direct natural-product-specific evidence in cancer is very limited. VRAC is best presented as an underexplored candidate mechanism rather than an established phytochemical target.	Hypothetical/underexplored	Demonstrate swelling-activated currents and LRRC8 dependence using pharmacological and genetic perturbation, with apoptosis assessed under controlled osmotic conditions.
Ca^2+^ influx and ER–mitochondria Ca^2+^ transfer	Excessive Ca^2+^ entry or ER-to-mitochondria transfer may induce mitochondrial ROS, depolarization, cytochrome-*c* release, and caspase activation.	Curcumin, resveratrol, quercetin, EGCG, berberine, capsaicin, and withaferin A have been associated with altered Ca^2+^ signaling and mitochondrial apoptosis in experimental models.	Moderate–strong mechanistic	Show that Ca^2+^ changes precede mitochondrial dysfunction and apoptosis and are attenuated by Ca^2+^ chelation or pathway-specific genetic/pharmacological rescue.
TRP-channel-linked phytochemical sensing	TRP channels can couple chemical sensing to Ca^2+^ influx, ROS generation, membrane-potential changes, and apoptosis.	Capsaicin–TRPV1 is the clearest example; curcumin, resveratrol, and quercetin have also been linked to TRP modulation, although causal evidence varies substantially by channel and model.	Moderate mechanistic; compound-specific	Demonstrate channel expression and activity and show that selective antagonism or genetic loss reduces both ion flux and apoptosis.
Store-operated Ca^2+^ entry through STIM1/Orai1	Store-operated Ca^2+^ entry supports cancer-cell proliferation and survival, but excessive or disrupted signaling may promote ER stress and mitochondrial apoptosis.	Curcumin, resveratrol, quercetin, EGCG, and berberine have been linked to Ca^2+^-dependent stress responses, but direct STIM1/Orai1 dependence is rarely established.	Emerging/mechanistic inference	Directly assess store-operated Ca^2+^ entry and demonstrate STIM1/Orai1 dependence using selective inhibition or genetic perturbation.
Mitochondrial ion channels and permeability-transition susceptibility	Mitochondrial Ca^2+^, K^+^, and permeability-transition pathways regulate membrane potential, ROS, swelling, and cytochrome-*c* release.	Curcumin, berberine, resveratrol, quercetin, EGCG, piperine, and withaferin A affect mitochondrial ion handling and apoptosis, although specific channel targets are often unresolved.	Moderate mechanistic	Distinguish general mitochondrial dysfunction from ion-channel-specific effects using direct measurements of mitochondrial ion flux and pathway-specific rescue.
Aquaporins and water movement	Aquaporins influence water permeability, migration, volume recovery, and apoptosis-associated shrinkage and may modify sensitivity to osmotic stress.	Curcumin, resveratrol, and other dietary compounds have been linked to aquaporin regulation, but evidence for a causal role in natural-product-induced cancer-cell apoptosis remains limited.	Emerging/underexplored	Demonstrate altered water permeability and volume kinetics and show that aquaporin inhibition or knockdown modifies apoptosis.
Plasma-membrane potential and electrogenic coupling	Cancer-associated changes in K^+^, Na^+^, Ca^2+^, and Cl^−^ conductance can alter Ca^2+^ entry, mitochondrial function, and apoptotic signaling.	Curcumin, resveratrol, quercetin, EGCG, berberine, and capsaicin may alter membrane potential or ion flux, but direct evidence for cancer-selective apoptosis through this mechanism is uneven.	Moderate mechanistic	Establish that membrane-potential changes precede death and are functionally linked to apoptosis through electrophysiological and rescue experiments.
Ion-channel dependence as a combination vulnerability	Partial disruption of ion and volume homeostasis may cooperate with redox stress, mitochondrial priming, ER stress, or membrane remodeling to convert adaptive stress into apoptosis.	Multi-target phytochemicals such as curcumin, EGCG, quercetin, resveratrol, berberine, capsaicin, sulforaphane, and withaferin A provide a rationale for testing ion–volume regulation as a co-determinant of response.	Hypothesis-generating	Use time-resolved multimodal studies combining ion-flux or cell-volume measurements with genetic channel perturbation and formal interaction analyses.

Note: Ion–volume dysregulation should be presented as an emerging and incompletely validated vulnerability layer, not as a mechanism established to the same degree as redox imbalance or mitochondrial priming. Ion flux, membrane potential, osmotic adaptation, and water movement may contribute to apoptotic commitment, but they may also arise secondarily after mitochondrial or caspase activation. Causality is most convincingly supported when ion or volume changes precede apoptosis, are directly measured, are modified by pharmacological or genetic rescue, and occur preferentially in cancer cells compared with relevant non-malignant controls. Primary membrane lysis and nonspecific osmotic injury should be excluded through membrane-integrity, hemolysis, and time-course analyses. Evidence levels summarize the highest level of support currently available for each feature and should not be interpreted as applying uniformly to every phytochemical listed. Representative compounds are illustrative rather than exhaustive. Abbreviations: AVD, apoptotic volume decrease; EGCG, epigallocatechin gallate; LRRC8, leucine-rich repeat-containing protein 8; STIM1, stromal interaction molecule 1; TRP, transient receptor potential; VRAC, volume-regulated anion channel.

**Table 7:** Survival-pathway features that may convert natural-product-induced stress into apoptosis.

Survival-Pathway Vulnerability Feature	Biological Rationale	Representative Natural-Product Relevance	Evidence Level	Key Validation Criteria
PI3K/Akt/mTOR dependence	Cancer cells may rely on PI3K/Akt/mTOR signaling to maintain growth, metabolism, survival, autophagy control, and resistance to apoptosis. Suppression of this axis may expose mitochondrial or ER-stress vulnerability.	Curcumin, quercetin, EGCG, resveratrol, berberine, sulforaphane, ginsenosides, and pterostilbene have been reported to suppress this pathway in cancer models [1,2,3,4,5,6,7,8,9,10].	Strong preclinical	Demonstrate pathway suppression before apoptotic commitment and show functional rescue with constitutively active Akt/mTOR or pathway-specific modulation.
AMPK-mediated metabolic stress	AMPK can suppress anabolic growth and mTOR signaling during energetic stress, but it may promote either apoptosis or cytoprotection depending on context.	Resveratrol, berberine, curcumin, EGCG, pterostilbene, and selected ginsenosides modulate AMPK/mTOR signaling in preclinical models [3,5,6,8,9,10].	Moderate–strong mechanistic; context-dependent	Establish whether AMPK is pro-death or protective using genetic or pharmacological inhibition together with metabolic and apoptotic readouts.
MAPK balance: ERK survival versus JNK/p38 stress signaling	ERK commonly supports proliferation and survival, whereas sustained JNK or p38 activation may promote mitochondrial, death-receptor, or ER-stress-associated apoptosis.	Curcumin, quercetin, resveratrol, EGCG, berberine, pterostilbene, sulforaphane, and ginsenosides have been linked to MAPK modulation [1,2,3,4,5,6,7,8,9,10].	Strong preclinical	Define the timing and duration of ERK, JNK, and p38 responses and demonstrate pathway dependence through inhibitor or genetic rescue experiments.
NF-κB inflammatory-survival addiction	Chronic NF-κB activity can sustain anti-apoptotic, inflammatory, angiogenic, and therapy-resistance programs.	Curcumin, EGCG, quercetin, resveratrol, berberine, sulforaphane, withaferin A, and pterostilbene have been reported to inhibit NF-κB signaling [1,2,3,4,5,6,7,8,10,11,12].	Strong preclinical	Show that NF-κB inhibition precedes apoptosis and that restoration of NF-κB activity attenuates cell death.
STAT3 survival and immune-evasion signaling	Constitutive STAT3 signaling promotes survival, stem-like traits, immune evasion, angiogenesis, and resistance to apoptosis.	Curcumin, resveratrol, EGCG, berberine, quercetin, pterostilbene, withaferin A, and ginsenosides have been linked to STAT3 suppression [1,3,4,5,6,7,8,10,11,12].	Strong preclinical	Demonstrate STAT3 dependency using reporter assays and genetic rescue rather than relying only on reduced phospho-STAT3 expression.
Wnt/β-catenin dependence and stem-like survival	Wnt/β-catenin signaling supports proliferation, EMT, stemness, invasion, and resistance. Tumors dependent on β-catenin transcription may be sensitive to its disruption.	Curcumin, EGCG, quercetin, resveratrol, berberine, sulforaphane, pterostilbene, and ginsenoside-related preparations have been investigated in several cancer models [4,5,8,9,10,13,14,15].	Moderate–strong preclinical	Distinguish β-catenin-specific effects from general growth inhibition using transcriptional assays, genetic manipulation, and apoptosis measurements.
Autophagy–apoptosis crosstalk	Autophagy may protect cells during stress or, when excessive or blocked, contribute to apoptosis or mixed regulated cell-death phenotype.	Curcumin, resveratrol, berberine, EGCG, quercetin, sulforaphane, ginsenosides, and withaferin A have been linked to autophagy–apoptosis interactions [1,3,5,6,7,8,9,10,16].	Strong preclinical; context-dependent	Measure autophagic flux and determine whether autophagy inhibition increases or decreases apoptosis. LC3-II accumulation alone is insufficient.
ER-stress/unfolded-protein-response dependence	Tumors with high secretory or proteostatic burden may depend on adaptive UPR signaling. Persistent phytochemical-induced ER stress may shift this response toward CHOP/JNK/BH3-linked apoptosis.	Curcumin, resveratrol, quercetin, berberine, celastrol, withaferin A, EGCG, and selected ginsenosides have been associated with UPR activation and mitochondrial apoptosis [3,5,6,7,8,10,16,17].	Moderate–strong mechanistic	Demonstrate that inhibition of ER-stress signaling reduces downstream mitochondrial apoptosis using pathway-specific rescue.
Cell-cycle checkpoint dependence	Checkpoint activation may halt proliferation and create conditions for apoptosis when repair or survival mechanisms fail.	Curcumin, quercetin, EGCG, resveratrol, berberine, sulforaphane, pterostilbene, ginsenosides, and withaferin A induce cell-cycle arrest in multiple models [1,2,3,4,5,6,7,8,9,10,13].	Strong preclinical for arrest; moderate for apoptosis coupling	Separate cytostasis from apoptosis using time-course, cell-cycle, DNA-synthesis, and apoptotic assays, with checkpoint-specific rescue where feasible.
Death-receptor and extrinsic-apoptosis sensitization	Suppression of NF-κB, Akt, STAT3, or related survival nodes may lower resistance to TRAIL-, Fas-, or TNF-associated apoptosis.	Curcumin, quercetin, EGCG, resveratrol, berberine, sulforaphane, and withaferin A have been linked to death-receptor sensitization in selected models [1,2,3,4,5,6,7,8,11,12].	Moderate preclinical; combination-specific	Demonstrate receptor and caspase-8 dependence and distinguish direct extrinsic apoptosis from mitochondrial amplification.
Integrated adaptive-survival network dependence	Cancer cells often depend on interconnected PI3K/Akt/mTOR, NF-κB, STAT3, MAPK, Wnt, autophagy, and UPR pathways rather than on one isolated node.	Multi-target phytochemicals such as curcumin, resveratrol, EGCG, quercetin, berberine, sulforaphane, pterostilbene, ginsenosides, and withaferin A commonly modulate several pathways simultaneously [1,2,3,4,5,6,7,8,9,10,11,12,13,14,15,16,17].	Hypothesis-generating/systems-level preclinical	Use time-resolved multi-pathway profiling and single-node rescue to distinguish coordinated pathway dependence from nonspecific signaling collapse.

Note: Survival-pathway addiction should be interpreted as a pre-existing vulnerability state only when pathway activity provides adaptive buffering against phytochemical-induced stress. Suppression of PI3K/Akt/mTOR, AMPK, MAPK, NF-κB, STAT3, Wnt/β-catenin, autophagy, UPR, or cell-cycle regulators does not by itself establish selective apoptosis. Causality is best supported when pathway modulation precedes apoptotic commitment, is functionally tested through rescue or phenocopy experiments, and produces a stronger effect in cancer cells than in relevant non-malignant controls. Evidence levels summarize the highest level of support currently available for each vulnerability feature and should not be interpreted as applying uniformly to every phytochemical listed. Representative compounds are illustrative rather than exhaustive. Abbreviations: EGCG, epigallocatechin gallate; EMT, epithelial–mesenchymal transition; ER, endoplasmic reticulum; UPR, unfolded protein response.

**Table 8:** Evidence-based framework for interpreting and distinguishing major cellular outcomes following exposure to plant-derived natural products.

Cellular Outcome	Core Evidence Supporting Interpretation	Recommended Confirmatory Evidence	Evidence Insufficient by Itself
Apoptosis	Coordinated apoptotic morphology (cell shrinkage, chromatin condensation, membrane blebbing, and apoptotic body formation), together with temporally ordered mitochondrial outer-membrane permeabilization (where intrinsic apoptosis is proposed), cytochrome c release, executioner-caspase activation, and PARP cleavage.	Time-course analysis demonstrating that apoptotic morphology precedes membrane rupture, together with functional suppression by caspase inhibition or genetic perturbation of apoptotic regulators. Where intrinsic apoptosis is proposed, dependence on BAX/BAK or BCL-2-family signaling should be demonstrated whenever feasible.	Reduced metabolic viability, Annexin V/propidium iodide staining, ROS accumulation, mitochondrial depolarization, altered BAX/BCL-2 expression, or isolated caspase activation without morphological confirmation, temporal analysis, and pathway-specific rescue [127,128,129,130].
Ferroptosis	Iron-dependent phospholipid peroxidation accompanied by impaired GPX4-, glutathione-, or SLC7A11-mediated antioxidant defense and compatible cellular morphology.	Rescue by ferrostatin-1, liproxstatin-1, iron chelation, or restoration of GPX4-dependent lipid-peroxide detoxification; limited dependence on caspase inhibition.	ROS generation, malondialdehyde, 4-HNE accumulation, glutathione depletion, mitochondrial dysfunction, or lipid peroxidation without ferroptosis-specific rescue [131,132,133].
Necroptosis	Activation of the RIPK3–MLKL signaling axis, demonstrated by MLKL phosphorylation together with oligomerization and/or plasma membrane translocation, in cells expressing functional RIPK3 and MLKL.	Rescue by genetic depletion of RIPK3 or MLKL or by validated pathway-specific inhibitors with appropriate specificity controls.	LDH release, plasma membrane permeabilization, cell swelling, RIPK1 expression, or caspase-independent death without evidence of RIPK3-dependent MLKL activation or pathway-specific rescue [134,135,136,137,138].
Pyroptosis	Gasdermin cleavage generating the pore-forming N-terminal fragment, accompanied by membrane pore formation, cell swelling, plasma membrane permeabilization, and inflammatory signaling where biologically relevant. The upstream caspase and responsible gasdermin should be identified.	Genetic depletion or pharmacological inhibition of the relevant gasdermin or upstream inflammasome/caspase pathway resulting in attenuation of membrane permeabilization and inflammatory signaling.	LDH release, IL-1β secretion, caspase-1 activation, membrane rupture, or inflammatory signaling without demonstration of gasdermin cleavage, pore formation, or pathway-specific rescue [139,140,141,142].
Accidental necrosis or direct membrane lysis	Rapid loss of plasma membrane integrity with early uptake of impermeant dyes, LDH release, cell swelling, and organelle disruption occurring in the absence of preceding evidence for regulated cell-death signaling.	Persistence despite inhibition of apoptosis, ferroptosis, necroptosis, or pyroptosis. For membrane-active compounds, direct membrane injury should be supported by orthogonal membrane-specific assays (e.g., hemolysis, liposome permeabilization, cholesterol-depletion or cholesterol-repletion studies, membrane biophysics, or live-cell imaging).	Late membrane permeabilization following apoptosis, isolated LDH release, uptake of impermeant dyes at a single endpoint, or cytotoxicity observed only at supraphysiological concentrations without temporal or mechanistic analysis [127,143,144,145,146].
Cytostasis	Sustained suppression of cellular proliferation with preserved plasma membrane integrity and minimal evidence of cell death, demonstrated by direct cell counting, growth-rate metrics, DNA-synthesis assays, cell-cycle analysis, clonogenic assays, or longitudinal live-cell imaging.	Demonstration of reversible growth arrest following compound withdrawal or, alternatively, irreversible loss of clonogenic capacity by clonogenic assays, thereby distinguishing cytostasis from delayed cytotoxicity.	Reduced metabolic activity, decreased confluence, lower Ki-67 expression, altered cell-cycle distribution, or reduced ATP content without direct assessment of viable cell number, membrane integrity, or long-term proliferative capacity [127,147,148].
Mixed death phenotype	Independent mechanistic evidence supporting activation of two or more regulated cell-death pathways within the same experimental system, dose range, or temporal sequence, recognizing that pathway overlap and phenotypic plasticity are common biological responses.	Partial protection by pathway-specific genetic or pharmacological interventions individually, with greater rescue following combined pathway inhibition, ideally supported by time-resolved or single-cell analyses demonstrating sequential or concurrent activation of distinct death programs.	Concurrent detection of nonspecific stress-associated markers (e.g., ROS accumulation, Annexin V positivity, lipid peroxidation, caspase activation, or LDH release) without pathway-specific rescue, temporal resolution, or independent mechanistic validation [127,143,149].

Note: The framework provides practical minimum criteria rather than an exhaustive protocol. No single marker is sufficient to define a death modality. Rescue experiments should use validated concentrations and, where feasible, be supported by genetic perturbation because pharmacological inhibitors may have off-target effects. Secondary necrosis should be distinguished from accidental necrosis (direct membrane lysis) by temporal analysis demonstrating that apoptotic execution precedes membrane rupture. Annexin V positivity indicates phosphatidylserine exposure but is not specific for apoptosis. Mixed phenotypes should be reported when independent pathway-specific evidence supports more than one death mechanism. Abbreviations: 4-HNE, 4-hydroxynonenal; DNA, deoxyribonucleic acid; Ki-67, proliferation marker Ki-67; ROS, reactive oxygen species.

**Table 9:** Practical framework for interpreting and distinguishing major cellular outcomes following natural-product exposure.

Potential Source of Bias	Why It Matters	Recommended Validation
Chemical identity, purity and batch consistency	Incorrect compound assignment or variable extract composition may produce irreproducible biological activity.	Confirm identity by liquid chromatography–tandem mass spectrometry (LC-MS/MS) and/or nuclear magnetic resonance (NMR); determine purity by high-performance liquid chromatography (HPLC) or ultra-performance liquid chromatography (UPLC); standardize botanical authentication, voucher specimens, and independent production batches.
Chemical stability	Degradation during storage or incubation may alter biological activity or generate degradation products.	Evaluate stability under culture conditions using chromatographic analysis before and after incubation; monitor pH- and light-dependent degradation where appropriate.
Colloidal aggregation	Self-assembled aggregates can nonspecifically inhibit proteins and generate false-positive activity.	Assess concentration dependence; evaluate detergent sensitivity; confirm activity after aggregate-disrupting conditions or orthogonal assays.
Pan-assay interference compounds (PAINS) and assay interference	Promiscuous compounds may interfere with multiple assay platforms independently of the intended biological target.	Screen for known interference chemotypes; confirm activity using orthogonal assays and independent readouts; avoid conclusions based on a single assay platform.
Redox cycling and nonspecific covalent reactivity	Apparent target inhibition may result from oxidative chemistry or indiscriminate protein modification rather than selective pharmacology.	Use antioxidant rescue controls where appropriate; evaluate reversibility and target specificity; verify target engagement by independent methods.
Optical interference	Intrinsic fluorescence, absorbance, or quenching may distort colorimetric or fluorometric assays.	Include cell-free controls; validate findings using alternative detection technologies and orthogonal biological assays.
Extract contamination and compositional variability	Minor constituents, contaminants, or extraction-dependent variability may account for observed biological activity.	Use standardized extraction procedures; characterize chemical fingerprints; compare independently prepared batches and, where possible, purified constituents.
Active metabolite formation	The biologically active species may differ from the administered parent compound.	Characterize metabolite profiles using pharmacokinetic studies or LC-MS/MS and evaluate biological activity of major metabolites where feasible.
Orthogonal confirmation	Single-assay conclusions are vulnerable to assay-specific artifacts.	Confirm activity using independent biological endpoints, target-engagement assays, genetic perturbation, or chemically unrelated validation methods before assigning mechanism.

Abbreviations: HPLC, high-performance liquid chromatography; LC-MS/MS, liquid chromatography–tandem mass spectrometry; NMR, nuclear magnetic resonance; PAINS, pan-assay interference compounds; UPLC, ultra-performance liquid chromatography.

## Data Availability

No new data were created or analyzed in this study. Data sharing is not applicable to this article.

## Abbreviations

The following abbreviations are used in this manuscript:

4-HNE	4-Hydroxynonenal
Annexin V/PI	Annexin V/propidium iodide
ARE	Antioxidant response element
ATP	Adenosine triphosphate
ATP/AMP	Adenosine triphosphate/adenosine monophosphate ratio
AVD	Apoptotic volume decrease
BAPTA-AM	1,2-Bis(o-aminophenoxy)ethane-N,N,N′,N′-tetraacetic acid acetoxymethyl ester
BrdU	Bromodeoxyuridine
C11-BODIPY	Lipid-peroxidation-sensitive boron-dipyrromethene fluorescent probe
CRISPR	Clustered regularly interspaced short palindromic repeats
DCPIB	4-(2-Butyl-6,7-dichloro-2-cyclopentyl-indan-1-on-5-yl)oxybutyric acid
DMAPT	Dimethylamino-parthenolide
DPH	1,6-Diphenyl-1,3,5-hexatriene
ECAR	Extracellular acidification rate
EdU	5-Ethynyl-2′-deoxyuridine
EGCG	Epigallocatechin gallate
EGTA	Ethylene glycol-bis(β-aminoethyl ether)-N,N,N′,N′-tetraacetic acid
EMT	Epithelial–mesenchymal transition
ER	Endoplasmic reticulum
FRAP	Fluorescence recovery after photobleaching
GC–MS	Gas chromatography–mass spectrometry
GSH	Reduced glutathione
GSH/GSSG	Reduced-to-oxidized glutathione ratio
HPLC	High-performance liquid chromatography
HPLC-DAD	High-performance liquid chromatography with diode-array detection
HPLC-UV	High-performance liquid chromatography with ultraviolet detection
IC_50_	Half-maximal inhibitory concentration
JC-1	5,5′,6,6′-Tetrachloro-1,1′,3,3′-tetraethylbenzimidazolylcarbocyanine iodide
LC–MS/MS	Liquid chromatography–tandem mass spectrometry
MDA	Malondialdehyde
MDR	Multidrug resistance
MitoSOX	Mitochondrial superoxide indicator
MitoTEMPO	Mitochondria-targeted superoxide dismutase mimetic antioxidant
MOMP	Mitochondrial outer-membrane permeabilization
MQAE	*N*-(Ethoxycarbonylmethyl)-6-methoxyquinolinium bromide
mPTP	Mitochondrial permeability-transition pore
NAC	*N*-Acetylcysteine
NADPH	Reduced nicotinamide adenine dinucleotide phosphate
NADPH/NADP^+^	Reduced-to-oxidized nicotinamide adenine dinucleotide phosphate ratio
NMR	Nuclear magnetic resonance
OCR	Oxygen-consumption rate
OCR/ECAR	Oxygen-consumption-rate-to-extracellular-acidification-rate relationship
PI	Propidium iodide
PPP	Pentose phosphate pathway
PS	Phosphatidylserine
ROS	Reactive oxygen species
Ru360	Ruthenium 360
RVD	Regulatory volume decrease
siRNA	Small interfering RNA
SOCE	Store-operated calcium entry
SYTOX	Membrane-impermeant nucleic-acid stain used to assess membrane permeability
TMA-DPH	1-(4-Trimethylammoniumphenyl)-6-phenyl-1,3,5-hexatriene
TMRE	Tetramethylrhodamine ethyl ester
TMRM	Tetramethylrhodamine methyl ester
TUNEL	Terminal deoxynucleotidyl transferase dUTP nick-end labelling
UPLC–MS	Ultra-performance liquid chromatography–mass spectrometry
UPR	Unfolded protein response
